# Biphen[*n*]arenes†
†Electronic supplementary information (ESI) available: Detailed synthetic procedures and characterization, crystal data for **EtBP3** and **EtBP4**, ESI spectra, additional ^1^H NMR spectra, Job plots, and determination of the association constants. CCDC 1016929 and 1016930. For ESI and crystallographic data in CIF or other electronic format see DOI: 10.1039/c4sc02422b


**DOI:** 10.1039/c4sc02422b

**Published:** 2014-09-17

**Authors:** Huanqing Chen, Jiazeng Fan, Xiaoshi Hu, Junwei Ma, Shilu Wang, Jian Li, Yihua Yu, Xueshun Jia, Chunju Li

**Affiliations:** a Department of Chemistry , Shanghai University , Shanghai , 200444 , P. R. China . Email: cjli@shu.edu.cn; b Shanghai Key Laboratory of Magnetic Resonance , Department of Physics , East China Normal University , Shanghai , 200062 , P. R. China; c Beijing National Laboratory for Molecular Sciences (BNLMS) , Beijing , 100190 , P. R. China

## Abstract

To design and exploit novel macrocyclic synthetic receptors is a permanent and challenging topic in supramolecular chemistry. Here we describe the one-pot synthesis, unique geometries and intriguing host–guest properties of a new class of supramolecular macrocycles – biphen[*n*]arenes (*n* = 3, 4), which are made up of 4,4′-biphenol or 4,4′-biphenol ether units linked by methylene bridges at the 3- and 3′- positions. The biphenarene macrocycles are conveniently accessible/modifiable and extremely guest-friendly. Particularly, biphen[4]arene is capable of forming inclusion complexes with not only organic cationic guests but also neutral π-electron deficient molecules. Compared with calixarenes, resorcinarenes, cyclotriveratrylenes and pillararenes with substituted mono-benzene units, the biphen[*n*]arenes reported here possess significantly different characteristics in both their topologic structures and their recognition properties, and thus can find broad applications in supramolecular chemistry and other areas.

## Abstract 2

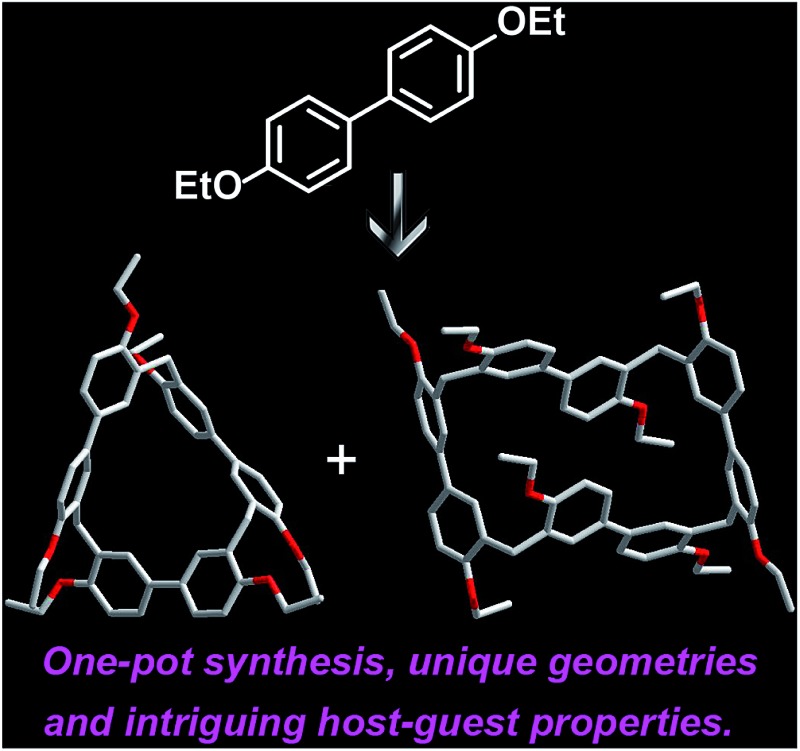
We describe the one-pot synthesis, unique geometries and intriguing host–guest properties of a new class of supramolecular macrocycles – biphen[*n*]arenes (*n* = 3, 4).

## Introduction

**Scheme 1:**
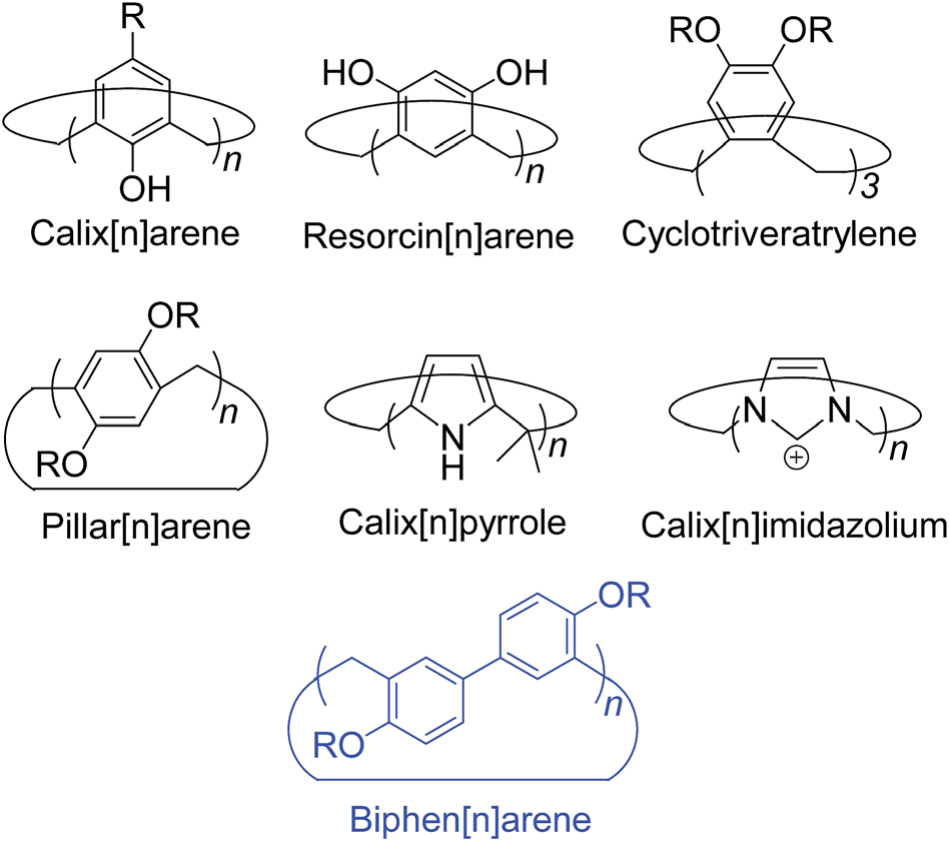
Structures of some typical macrocyclic arenes.

Macrocyclic synthetic receptors bearing preorganized cavities and multivalent binding sites have played a vital role in the birth of modern supramolecular chemistry and its rapid development.^1,2^ To design and exploit novel macrocyclic hosts with unique structures and good host–guest properties is a permanent and challenging topic in this area. Some new molecular containers recently reported include Sessler's “Texas-sized” box,^3^ Chun and Singh's calix[4]imidazolium,^4^ Ogoshi's pillar[5]arene,^5^ Stoddart's Ex-box,^6^ Sindelar's bambus[6]uril,^7^ Flood's cyanostar,^8^ and others.^9,10^ Among the supramolecular hosts, macrocyclic arenes based on methylene linked aromatic rings have been the focus of considerable recent research. Starting from calixarenes, the third generation of supramolecular hosts, a series of their structurally similar scaffolds have also been developed, displaying different geometries and molecular recognition/self-assembly behaviors (Scheme 1).^4,5,11–13^ For example, calixpyrroles, calixpyridines and the recently reported caliximidazoliums show considerable promise in the area of anion complexation and sensing.^4,11^ Cyclotriveratrylenes have been utilized for the complexation and separation of fullerenes.^12,14^ Pillararenes with symmetrical pillar architectures have exhibited novel binding abilities towards neutral guests.^15^ Furthermore, these macrocycles have also seeded many potential applications in biology, and materials and environmental science such as drug delivery,^16^ extraction and separation,^14,17^ stimuli responsive materials,^18^ and artificial transmembrane channels.^19^

Hitherto, most of the macrocyclic arenes have been based on mono-benzene and mono-heterocycle units (Scheme 1). Typical supramolecular macrocycles consisting of substituted benzene monomers include calixarene from phenol, resorcinarene from resorcinol, cyclotriveratrylene from veratrole, and pillararene from hydroquinone. Additionally, calixnaphthalenes^20^ have also been demonstrated, but they have not gained as much attention because (i) they do not show good cavity host–guest properties; (ii) their structures are not novel and are similar to calixarenes; and (iii) their synthesis is not easy and usually needs multi-step reactions.

Herein, we report the synthesis, structures, and molecular binding behavior of a new family of macrocyclic arenes, which are made up of 4,4′-biphenol or 4,4′-biphenol ether units linked by methylene bridges at the 3- and 3′- positions (Scheme 1). According to the naming convention of resorcin[4]arenes (based on resorcinol monomers), this new family of supramolecular macrocycles is named as biphen[*n*]arenes. The biphenarene hosts designed here could be conveniently achieved by a one-pot Lewis acid-catalyzed condensation from commercial reagents, and they are expected, and have been found, to have extraordinary architectures and intriguing binding properties.

## Results and discussion

**Scheme 2:**
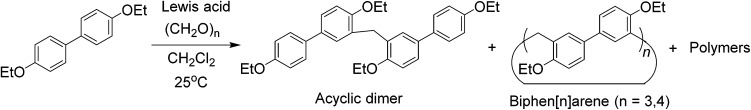
Synthesis of per-ethylated biphen[3,4]arenes.

**Fig. 1:**
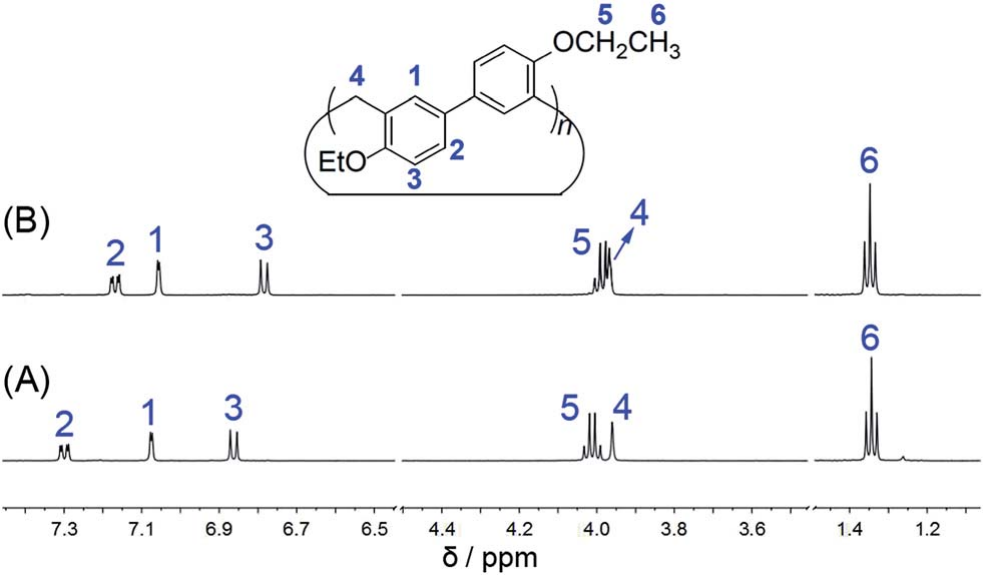
^1^H NMR spectra (500 MHz, 298 K) of **EtBP3** (A) and **EtBP4** (B) at 3.0 mM in CD_2_Cl_2_.

For the purpose of easy preparation, a direct cyclization strategy but not a fragment coupling approach was preferentially adopted in the present studies. It was found that the reactions of 4,4′-biphenol diethyl ether and paraformaldehyde (or formaldehyde aqueous solution) in the presence of a strong base (*e.g.* NaOH and KOH) or strong acid (*e.g.* HCl, H_2_SO_4_ and CF_3_COOH) could not give any cyclic oligomers. Nevertheless, when using a Lewis acid, such as FeCl_3_, BF_3_·O(Et)_2_, and trifluoromethanesulfonic acid (TfOH), as the catalyst, the reactions proceeded smoothly, and one acyclic dimer and two cyclic oligomers containing 3 and 4 biphenol diethyl ether units were successfully obtained (Scheme 2). After several attempts, BF_3_·O(Et)_2_ proved to be a little more efficient than FeCl_3_ and TfOH. After optimisation of the reaction conditions in the presence of BF_3_·O(Et)_2_ with respect to reaction solvent, temperature, and catalyst amount, the acyclic dimer (BPD), per-ethylated biphen[3]arene (**EtBP3**) and biphen[4]arene (**EtBP4**) were prepared in 9%, 22% and 8% yields, respectively. Furthermore, another larger macrocycle with a *m*/*z* value corresponding to the cyclic pentamer was also detected in the high-resolution mass spectrometry (HRMS) experiments of the reaction mixture. However, its yield was so poor that it was not successfully isolated for further characterization. The reaction time significantly affects the product yields. It was found that the best reaction time was 1.5–2 hours; further extending the time decreased the yields of both **EtBP3** and **EtBP4** and increased the yield of the polymeric product. For example, after 24 hours, the yields of **EtBP3** and **EtBP4** were only 5% and 1%. Furthermore, the condensation of 4,4′-biphenol dimethyl ether was also examined. Similarly, the acyclic dimer (yield: 12%), cyclic trimer (per-methylated biphen[3]arene, **MeBP3**, yield: 24%) and cyclic tetramer (per-methylated biphen[4]arene, **MeBP4**, yield: 5%) were successfully prepared. It should be pointed out that the distributions of the cyclic trimer and the tetramer were totally different in the syntheses of the ethylated and methylated biphenarenes, with yield ratios for trimer : tetramer of 2.8 : 1 and 4.8 : 1 respectively. **EtBP3** and **EtBP4** were characterized by ^1^H NMR, ^13^C NMR, and HRMS, as well as by their melting points. They exhibit similar ^1^H NMR and ^13^C NMR spectra, as shown in Fig. 1 and S1–S4.†

**Scheme 3:**
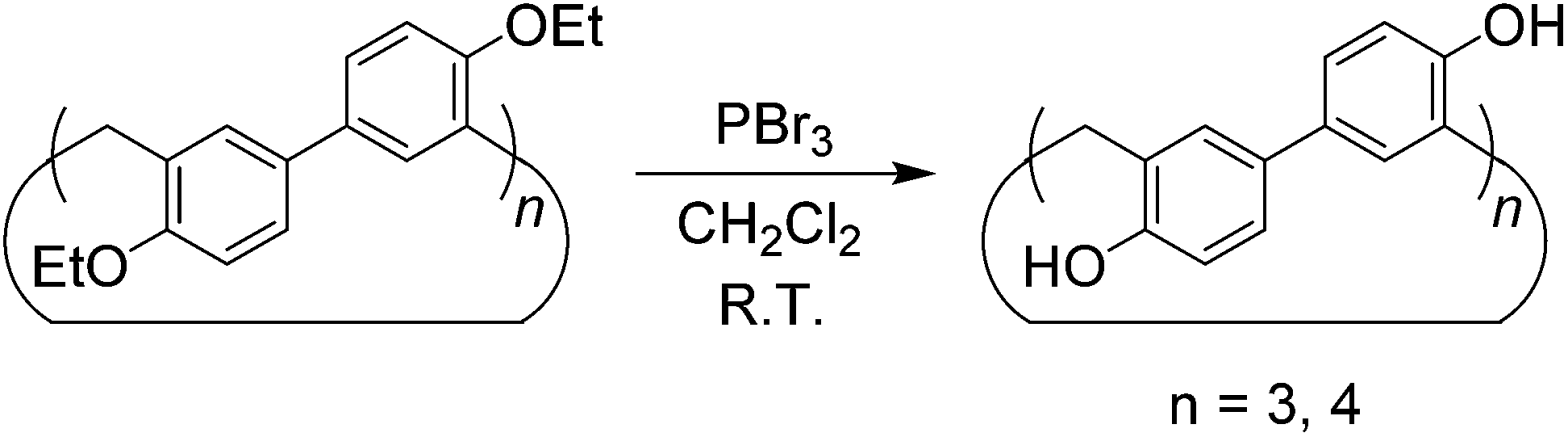
Synthesis of per-hydroxylated biphen[3,4]arenes.

Although the cyclotrimer and cyclotetramer were formed in only moderate yields (30% overall yield), the biphenarene hosts are intrinsically easy to prepare since they can be obtained by a one-step condensation reaction using commercial reagents. It is well documented that the modification of supramolecular hosts by attaching various peripheral functional groups can provide further interesting properties and functionalities. As depicted in Scheme 3, the cleavage of the ether groups in **EtBP3** and **EtBP4** by reaction with excess BBr_3_ in CH_2_Cl_2_ could quantitatively produce per-hydroxylated biphen[3,4]arenes (**OHBP3** and **OHBP4**). Therefore, it will be straightforward to prepare functionalized biphenarene derivatives through nucleophilic substitution reactions between **OHBP3**/**OHBP4** with alkylating agents in the presence of a suitable base. Besides the hydroxyl groups, the benzene rings of biphenarenes should also be reactive sites. That is to say, biphenarene hosts can be not only easy to prepare, but also facilely chemically modified. This is certainly significant for the further construction of efficient recognition/assembly systems and extending the applications of this new family of macrocycles.

**Fig. 2:**
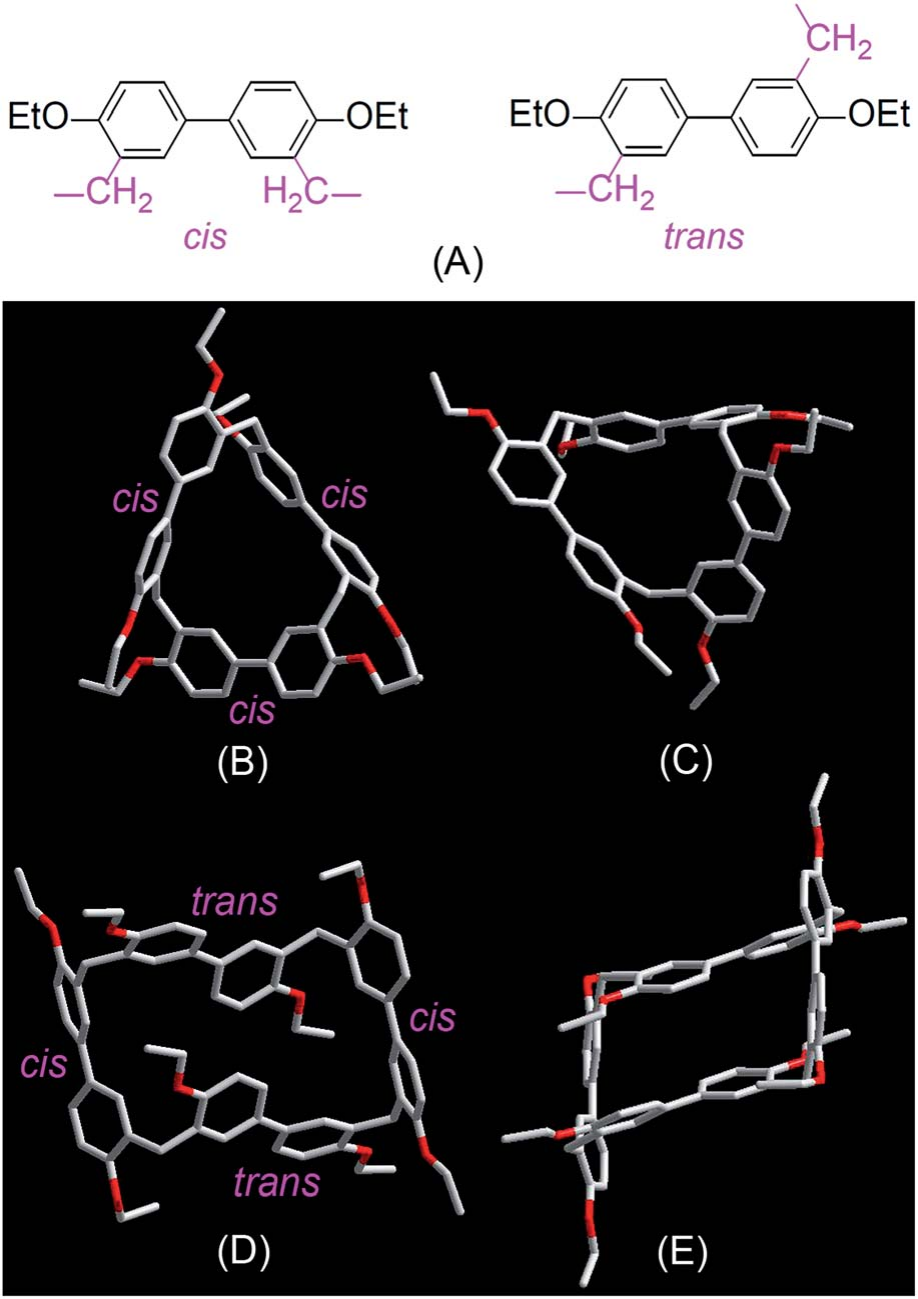
*Cis*- and *trans*-conformation of biphenyl monomers in biphenarenes (A) and crystal structures of **EtBP3** (B and C) and **EtBP4** (D and E).

Single crystals of **EtBP3** and **EtBP4** suitable for X-ray analysis were grown by slow evaporation of their CH_2_Cl_2_–*n*-hexane solutions at room temperature. As can be seen from Fig. 2, their structures are completely different. **EtBP3** exhibits a distorted triangular-prism structure and does not have an effective cavity in the solid state (Fig. 2B and C). **EtBP4** has a cuboid-like structure and exists in the form of a ‘partial chair’ topology, which is similar to that of the “Texas-sized” box.^3^ The biphen[4]arene molecular container can be regarded as a new type of neutral molecular box with π-electron rich cavities, which could complement electron-deficient tetracationic boxes such as Stoddart's “blue box”^21^ and Sessler's “Texas-sized” box.^3^ It is also interesting to note that the biphenyl units in biphenarenes could exist in two conformations, *i.e.*, a *cis*- and *trans*-conformation according to the relative position of the two methylene linkers (Fig. 2A). While all of the three biphenyl units in **EtBP3** are in the *cis*-conformation (Fig. 2B and C), in **EtBP4** two of the biphenyl units are in the *cis*-conformation and two are in the *trans*-conformation which are positioned in an alternating manner (Fig. 2D and E).

**Scheme 4:**
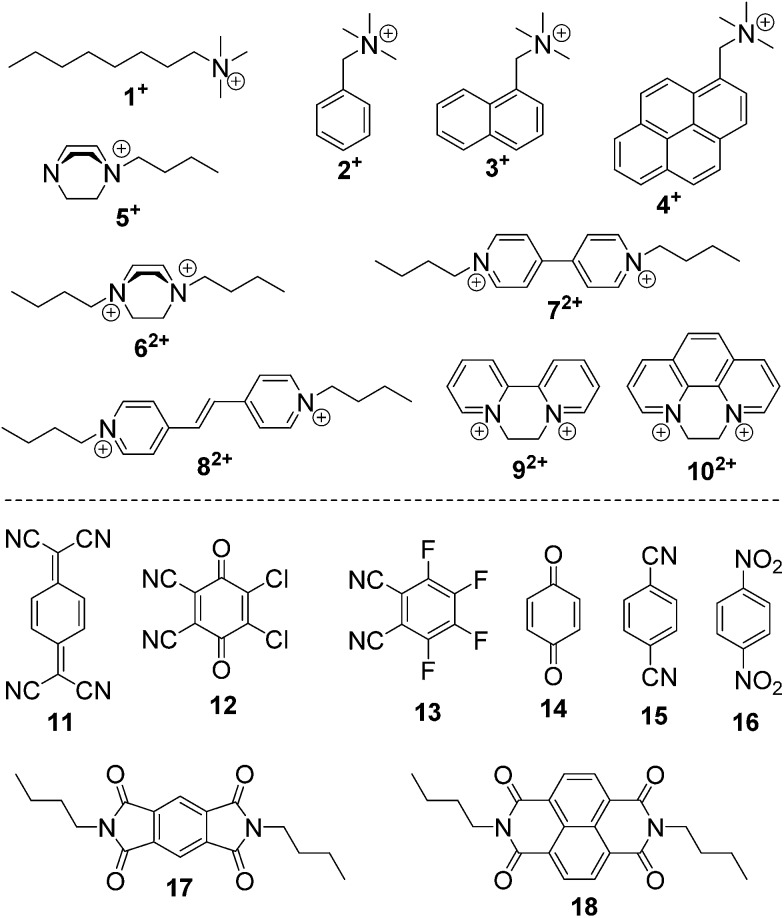
Structures of cationic and neutral guests.

**Fig. 3:**
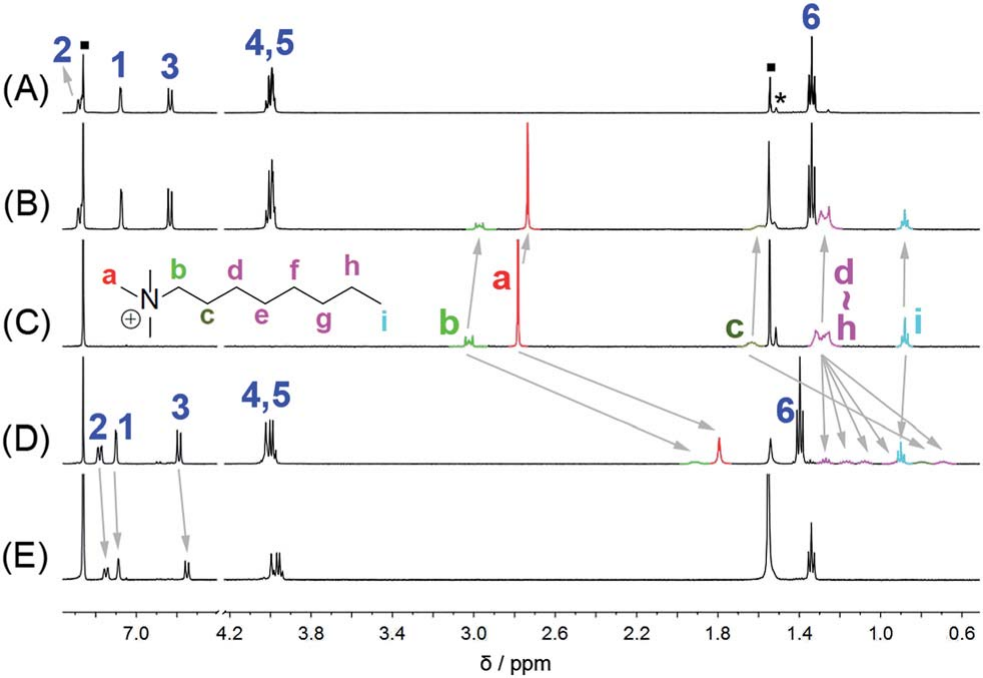
^1^H NMR spectra (500 MHz, 298 K) of (A) **EtBP3**, (B) **1**·BArF + **EtBP3**, (C) **1**·BArF, (D) **1**·BArF + **EtBP4**, and (E) **EtBP4** in CDCl_3_ at 2.9–3.2 mM. “■” = solvent/water; “*” = solvent impurities.

The host–guest chemistry of **EtBP3** and **EtBP4** was then investigated. Due to their π-electron rich characteristics, a series of organic cationic molecules (**1^+^–10^2+^**) and neutral π-electron deficient molecules (**11–18**) were chosen as guests (Scheme 4). Fig. 3 shows the ^1^H NMR spectra of *n*-octyltrimethyl ammonium tetrakis[3,5-bis(trifluoromethyl)phenyl] borate (**1**·BArF) in CDCl_3_ recorded in the absence and in the presence of approximately 1.0 equiv. of the **EtBP3**/**EtBP4** hosts. It is found that in the presence of **EtBP4**, the proton signals of **1^+^** derived from the methyl H_a_ and methylenes exhibit very pronounced upfield displacements (for example, Δ*δ* = –0.99 and –1.11 ppm for H_a_ and H_b_) and broadening as a consequence of inclusion-induced shielding effects (Fig. 3D). Meanwhile, the signal corresponding to the tail methyl (H_i_) shifts slightly downfield (Δ*δ* = 0.02 ppm), which is characteristic of the protons being located just outside the host's cavity portal.^22^ On the other hand, the host is deshielded by the presence of the guest, since the proton signals of **EtBP4** display downfield displacement (Δ*δ* = 0.01–0.04 ppm). The binding induced NMR changes are consistent with the formation of an interpenetrated complex. In contrast, upon the addition of **EtBP3**, no obvious signal changes can be observed for H_c–i_ of **1**·BArF, and the head H_a_ and H_b_ show relatively small upfield shifts (–0.05 ppm). These results indicate the formation of a shallow inclusion complex with the guest's “^+^NMe_3_” site. This is reasonable since **EtBP3** does not possess an effective cavity (Fig. 2B and C). The formation of 1 : 1 **1**·BArF⊂**EtBP3**/**EtBP4** complexes was further confirmed by electrospray ionization (ESI) mass spectroscopy experiments (Fig. S38†) and Job plots (Fig. S39†). In the ESI mass spectrum of an equimolar mixture of **1**·BArF and **EtBP3** (or **EtBP4**), only one intense peak for the 1 : 1 complex [**1**⊂**EtBP3**]^+^ with *m*/*z* 934.6 (or [**1**⊂**EtBP4**]^+^ with *m*/*z* 1188.7) was observed. By employing ^1^H NMR titration experiments, the association constants (*K*_a_) for these two complexes were determined. As expected, the *K*_a_ value of **1**·BArF⊂**EtBP4** ((1.3 ± 0.1) × 10^3^ M^–1^) is much larger than that for **EtBP3** (28 ± 2 M^–1^) due to their completely different binding characteristics.

For another three quaternary ammonium salts **2**·BArF–**4**·BArF, similar complexation modes were found, *i.e.*, they formed interpenetrated [2]pseudorotaxane-type complexes with **EtBP4**, but shallow inclusion complexes with **EtBP3**. All four quaternary ammoniums give similar *K*_a_ values upon complexation with **EtBP3** because their binding sites are “^+^NMe_3_” moieties and the substituents do not affect the host–guest affinities. However, for **EtBP4**, the binding abilities are closely related to the substituted groups since they are engulfed by the host. The *K*_a_ values of **EtBP4** with **3**·BArF and **4**·BArF with larger naphthyl and pyrenyl moieties are 3.9 and 3.7 times larger than that for **2**·BArF with a phenyl group. This may be attributed to the size-fit effects between the guests and **EtBP4**; larger naphthyl and pyrenyl groups are relatively suitable for the **EtBP4** cavity, leading to large association constants. Meanwhile, **1^+^** with an *n*-octyl group exhibits a stronger affinity than **2^+^**, possibly because the flexible octyl group could twist to fit the host cavity.

Among the cationic guests, **5**·BArF and **6**·2BArF bearing 1,4-diazabicyclo [2.2.2] octane (DBO) cations exhibit the largest binding constants with **EtBP4** (of the magnitude of 10^4^ M^–1^, Table 1), suggesting that spherical DBO moieties are excellent matches in size and shape with **EtBP4**'s cavity (Fig. S40 and S41†). **EtBP4** can also form [2]pseudorotaxane-type complexes with pyridinium-based dicationic guests **7**·2BArF–**10**·2BArF (Fig. S43 and S46†); the larger guests **9^2+^** and **10^2+^** show stronger binding strengths than **7^2+^** and **8^2+^** (Table 1). Although **EtBP3** cannot bind with **7^2+^** and **8^2+^** (Fig. S42†), it can form shallow inclusion complexes with **9^2+^** and **10^2+^** in such a way that the main binding site for the host is the “^+^N(CH_2_)_2_N^+^” part (Fig. S44†), which are similar to the complexes formed with the quaternary ammonium guests **1**·BArF–**4**·BArF.

**Table 1:** *K*_a_ values^*a*^ for 1 : 1 complexation of the guests with **EtBP3**/**EtBP4** at 298 K

Guest	Solvent^*b*^	**EtBP3**	**EtBP4**
**1^+^**	CDCl_3_	28 ± 2	(1.3 ± 0.1) × 10^3^
**2^+^**	CDCl_3_	29 ± 1	570 ± 40
**3^+^**	CDCl_3_	28 ± 2	(2.2 ± 0.3) × 10^3^
**4^+^**	CDCl_3_	26 ± 3	(2.1 ± 0.2) × 10^3^
**5^+^**	CDCl_3_	^*c*^	(1.5 ± 0.3) × 10^4^
**6^2+^**	CD_2_Cl_2_	^*c*^	(3.1 ± 0.4) × 10^4^
**7^2+^**	CD_2_Cl_2_	^*c*^	92 ± 5
**8^2+^**	CD_2_Cl_2_	^*c*^	41 ± 6
**9^2+^**	CD_2_Cl_2_	34 ± 4	320 ± 30
**10^2+^**	CD_2_Cl_2_	39 ± 2	390 ± 10
**11**	CDCl_3_	^*c*^	61 ± 12
**12**	CDCl_3_	^*c*^	100 ± 20
**13–18**	CDCl_3_	^*c*^	^*c*^

^*a*^The *K*_a_ values were determined by NMR titration methods.

^*b*^Dicationic guests **6^2+^–10^2+^** are not soluble in CDCl_3_, so their *K*_a_ values with the hosts were determined in CD_2_Cl_2_.

^*c*^No interactions were found or at least the association constants were too small (<10 M^–1^) to be accurately calculated.

The complexation of **EtBP3** and **EtBP4** towards a series of neutral π-electron deficient molecules, **11–18** (Scheme 4), was then examined. In the presence of **EtBP4**, the peak for the aromatic protons (H_a_) of 7,7,8,8-tetracyanoquinodimethane (TCNQ, **11**) displays substantial upfield shifts (Δ*δ* = –0.20 ppm) and broadening effects compared to the free guest as a consequence of inclusion-induced shielding effects (Fig. S47†). In contrast, no obvious NMR changes were observed when mixing **11** and **EtBP3** (Fig. S46†). These observations reveal that **11** could form an inclusion complex with the larger **EtBP4**, but cannot bind with the smaller **EtBP3**. Although 2,3-dichloro-5,6-dicyano-1,4-benzoquinone (DDQ, **12**) does not have proton signals, its complexation with **EtBP4** can be detected according to the signal changes of the host (Fig. S48A†). For the other six neutral guests, **13–18**, no effective host–guest interactions with either **EtBP3** or **EtBP4** were found (Fig. S48–S50†). Although the corresponding *K*_a_ values are relatively low, 61 ± 12 M^–1^ for **11**⊂**EtBP4** and 100 ± 20 M^–1^ for **12**⊂**EtBP4**, the complexation of neutral guests are interesting since calixarenes usually cannot form such complexes in organic solutions.

## Conclusions

In summary, we have presented a new family of macrocyclic receptors, biphen[*n*]arenes (*n* = 3,4). They are made up of 4,4′-biphenol or 4,4′-biphenol ether units linked by methylene bridges at the 3- and 3′- positions. The biphenarene hosts reported here have the following intrinsic characteristics and properties:

(i) easy accessibility: per-ethylated biphen[3,4]arenes were prepared through a one-step condensation using commercial reagents; the deprotection of the ethoxy moieties in per-ethylated biphen[3,4]arenes quantitatively produced per-hydroxylated biphen[3,4]arenes;

(ii) convenient modification: it should be possible to conveniently chemically modify them due to their reactive hydroxyl groups and benzene rings;

(iii) unique geometries: their topologic structures are completely different to macrocyclic arenes based on mono-benzene units such as calixarenes, resorcinarenes, cyclotriveratrylenes and pillararenes;

(iv) excellent cavity host–guest properties: biphen[4]arene is extremely guest-friendly, and is capable of binding both cationic guests and neutral molecules to form inclusion complexes.

Considering the above aspects, this new family of hosts is expected to be applicable in a variety of supramolecular systems such as rotaxanes, catenanes and polymeric aggregates. Furthermore, it is easy to afford their functionalized derivatives, making them promising candidates for applications in chemosensors, nanomaterials, ion and molecule transport, supramolecular amphiphiles, and *etc.* We believe that biphenarene chemistry will be quite prosperous in the near future.
