# Supplementary material for: Biphen[n]arenes
Source: Chem Sci. 2014 Sep 17;6(1):197–202. doi: 10.1039/c4sc02422b (PMC5424672; doi:10.1039/c4sc02422b)
Supplement: Supplementary file 1 [file SC-006-C4SC02422B-s001.pdf]

Electronic Supplementary Material (ESI) for Chemical Science.
This journal is © The Royal Society of Chemistry 2014

Electronic Supplementary Information

Biphen[*n*]arenes

Huanqing Chen,[a] Jiazeng Fan,[a] Xiaoshi Hu,[b] Junwei Ma,[a] Shilu Wang,[a] Jian Li,[a] Yihua Yu,[b] Xueshun Jia[a] and Chunju Li*[a,c]

[a] *Department of Chemistry, Shanghai University, Shanghai, 200444, P. R. China.*

[b] *Shanghai Key Laboratory of Magnetic Resonance, Department of Physics, East China Normal University, Shanghai, 200062, P. R. China.*

[c] *Beijing National Laboratory for Molecular Sciences (BNLMS), Beijing, 100190, P. R. China.*

E-mail: cjli@shu.edu.cn.

Contents

Experimental section. S2

Crystal data of EtBP3 and EtBP4. S9

Copies of ^{1}H NMR and ^{13}C NMR spectra of hosts and guests. S11

ESI mass spectra of **1**•BArF⊂EtBP3 and **1**•BArF⊂EtBP4 complexes. S30

Job plots. S31

^{1}H NMR spectra of guests in the absence and presence of EtBP3 and EtBP4 hosts. S32

Determination of the association constants. S42

References. S43

Experimental section.

Materials and methods.

Sodium tetrakis[3,5-bis(trifluoromethyl)phenyl]borate (NaBArF) was prepared according to literature procedures.[S1] Organic cationic guests **1**$^{+}$−**10**$^{2+}$ with BArF^{-} counter anions were prepared from their bromide salts using our previously reported methods.[S2] Neutral guests **11**−**16** were commercially available and used as received. **17** and **18** were prepared by literature methods and recrystallized and dried under reduced pressure before use.[S3] Melting points were obtained on an X-4 digital melting point apparatus without correction. ^{1}H NMR and ^{13}C NMR spectra were recorded on a Bruker AV500 instrument. High-resolution mass spectra (HRMS) were recorded on a Bruker Daltonics, Inc. APEXIII 7.0 TESLA FTMS instrument. ESI Mass spectra were performed on a Thermofinnigan LCQ Advantage LC-MS.

Synthesis of EtBP3 and EtBP4.

To the solution of 4,4'-biphenol diethyl ether (2.4 g, 10 mmol) in dichloromethane (120 mL) was added paraformaldehyde (0.31 g, 10 mmol). Boron trifluoride diethyl etherate (2.5 ml, 20 mmol) was then added to the reaction mixture. The mixture was stirred at 25 ^{o}C for 2 hours. Then the reaction was quenched by addition of 60 mL water. The organic phase was separated and washed with saturated aqueous $NaHCO_3$, and brine. The organic layer was dried over anhydrous Na_2SO_4 and concentrated. The residue was purified by column chromatography on silica gel (eluent: 3/1 v/v *n*-hexane/CH_2Cl_2 gradually changing to 1/1 v/v *n*-hexane/CH_2Cl_2) to afford acyclic dimer BPD (0.22 g, 9%), cyclic trimer EtBP3 (0.56 g, 22%), and cyclic tetramer EtBP4 (0.20 g, 8%) as white solids.

BPD. m.p. 218−220 °C. ^{1}H NMR (500 MHz, $CDCl_3$): δ (ppm): 7.42 (dd, *J* = 7.0, 2.0 Hz 6H), 7.32 (dd, *J* = 8.5, 2.5 Hz 2H), 6.90 (dd, *J* = 6.5, 2.0 Hz 4H), 6.87 (d, *J* = 8.5 Hz, 2H), 4.08~4.03 (m, 10H), 1.43~1.39 (m, 12H). ^{13}C NMR (125 MHz, $CDCl_3$, 298 K): δ (ppm): 157.8, 156.2, 133.8, 132.8, 129.7, 129.2, 127.6, 125.1, 114.6, 111.5 (C of biphenyl), 63.7, 63.5 (C of methylene in ethoxy group), 29.7 (C of methylene bridge of BPD, 15.0, 14.9 (C of methyl in ethoxy group). HRMS (ESI): $C_{33}H_{36}O_4Na^+$, calcd *m/z* 519.2506; found *m/z* 519.2511.

EtBP3. m.p. 295−297 °C. ^{1}H NMR (500 MHz, CD_2Cl_2 , 298 K): δ (ppm): 7.30 (dd, *J* = 8.5, 2.5 Hz, 6H), 7.07 (d, *J* = 2.5 Hz, 6H), 6.86 (d, *J* = 8.5 Hz, 6H), 4.01 (q, *J* = 7.0 Hz 12H), 3.96 (s, 6H), 1.34 (t, *J* = 7.0 Hz 18H). ^{13}C NMR (125 MHz, $CDCl_3$, 298 K): δ (ppm): 155.9, 133.4, 130.0, 128.7, 125.1, 111.8 (C of biphenyl), 63.9 (C of methylene in ethoxy group), 28.9 (C of methylene bridge of EtBP3), 14.9 (C of methyl in ethoxy group). HRMS (ESI): $C_{51}H_{54}O_6Na^+$, calcd *m/z* 785.3813; found *m/z* 785.3833.

EtBP4. m.p. 245−246 °C. ^{1}H NMR (500 MHz, CD_2Cl_2 , 298 K): δ (ppm): 7.17 (dd, *J* = 8.5, 2.5 Hz, 8H), 7.06 (d, *J* = 2.5 Hz, 8H), 6.78 (d, *J* = 8.5 Hz,8H), 4.01~3.97 (m, 24H), 1.35 (t, *J* = 7 Hz 24H). ^{13}C NMR (125 MHz, $CDCl_3$, 298 K): δ (ppm): 155.8, 133.5, 129.6, 128.8, 125.2, 111.5 (C of biphenyl), 63.8 (C of methylene in ethoxy group), 29.2 (C of methylene bridge of EtBP4), 15.0 (C of methyl in ethoxy group). HRMS (ESI): $C_{68}H_{72}O_8Na^+$, calcd *m/z* 1039.5119; found *m/z* 1039.5117.

Synthesis of MeBP3 and MeBP4.

MeBP3 and MeBP4 were synthesized from 4,4'-biphenol dimethyl ether by using similar synthetic method for the preparation of EtBP3/EtBP4.

Acyclic dimer. Yield 12%. m.p. 204−206 °C. ^{1}H NMR (500 MHz, $CDCl_3$, 298 K): δ (ppm): 7.41 (dd, *J* = 6.5, 2.0 Hz 2H), 7.36 (m, 4H), 7.30 (d, *J* = 2.0 Hz 2H), 6.92 (m, 6H), 4.05 (s, 2H); 3.87 (s, 6H); 3.82 (s, 6H). ^{13}C NMR (125 MHz, $CDCl_3$, 298 K): δ (ppm): 158.7, 157.0, 133.9, 133.2, 129.4, 129.0, 127.8, 125.5, 114.2, 110.7 (C of biphenyl), 55.7, 55.4 (C of methyl), 30.3 (C of methylene bridge). HRMS (ESI): $C_{29}H_{28}O_4NH_4^+$, calcd *m/z* 458.2326; found *m/z* 458.2316.

MeBP3. Yield 24%. m.p. 325−326 °C. ^{1}H NMR (500 MHz, $CDCl_3$, 298 K): δ (ppm): 7.31 (dd, *J* = 8.5, 2.0 Hz, 6H), 7.06 (d, *J* = 2.0 Hz, 6H), 6.87 (d, *J* = 8.5 Hz, 6H), 3.97 (s, 6H), 3.80 (s, 18H). ^{13}C NMR (125 MHz, $CDCl_3$, 298 K): δ (ppm): 156.7, 133.6, 129.6, 128.8, 125.5, 110.9 (C of biphenyl), 55.9 (C of methyl), 29.1 (C of methylene bridge of MeBP3). HRMS (ESI): $C_{45}H_{42}O_6Na^+$, calcd *m/z* 701.2874; found *m/z* 701.2853.

MeBP4. Yield 5%. m.p. > 330 °C. ^{1}H NMR (500 MHz, CD_2Cl_2 , 298 K): δ (ppm): 7.19 (dd, *J* = 8.5, 2.5 Hz, 8H), 6.97 (d, *J* = 2.5 Hz, 8H), 6.80 (d, *J* = 8.5 Hz, 8 H), 3.91 (s, 8H), 3.77 (s, 24H); ^{13}C NMR (175 MHz, CD_2Cl_2, 298 K): δ (ppm): 155.8, 132.6, 128.1, 127.6, 124.5, 109.6 (C of biphenyl), 54.7 (C of methyl), 28.9 (C of methylene bridge of MeBP4). HRMS (ESI): $C_{60}H_{56}O_8Na^+$, calcd *m/z* 927.3867; found *m/z* 927.3874.

Synthesis of OHBP3 and OHBP4.

OHBP3. To a solution of EtBP3 (380 mg, 0.50 mmol) in anhydrous dichloromethane (20 mL) was added excess boron tribromide (2.5 g, 10 mmol). The reaction mixture was stirred at room temperature for 36 h. Then the mixture was poured into ice water. The resulting precipitate was collected by filtration and washed with cold water to give 290 mg (0.49 mmol) of OHBP3 quantitatively. m.p. >330 °C. ^{1}H NMR (500 MHz, acetone-d_6, 298 K): δ (ppm):

7.34 (d, J = 2.5 Hz, 6H), 7.25 (dd, J = 8.5, 2.5 Hz, 6H), 6.89 (d, J = 8.4 Hz, 6H), 3.98 (s, 6H). ^{13}C NMR (125 MHz, acetone-d_6, 298 K): δ (ppm): 153.2, 133.3, 128.5, 127.4, 125.5, 115.7 (C of biphenyl), 30.1 (C of methylene bridge of OHBP3). HRMS (ESI): $C_{39}H_{30}O_6Na^+$, calcd *m/z* 617.1935; found *m/z* 617.1931.

OHBP4. OHBP4 was synthesized from EtBP4 by using similar synthetic method for the preparation of OHBP3. m.p. >330 °C. ^{1}H NMR (500 MHz, acetone-d_6, 298 K): δ (ppm): 7.30 (s, 8H), 7.16 (dd, J = 8.0, 2.5 Hz, 8H), 6.84 (d, J = 8.0 Hz, 8H), 3.97 (s, 8H). ^{13}C NMR (125 MHz, acetone-d_6, 298 K): δ (ppm): 153.3, 133.2, 128.5, 127.4, 125.4, 115.5 (C of biphenyl), 30.0 (C of methylene bridge of OHBP4). HRMS (ESI): $C_{52}H_{40}O_8Na^+$, calcd *m/z* 815.2615; found *m/z* 815.2607.

Synthesis of cationic guests with BArF$^-$ counteranions.

Organic cationic guests $\mathbf{1}^+$–$\mathbf{10}^{2+}$ with BArF$^-$ counter anions were prepared from their bromide salts using our previously reported methods.[S2]

1·BArF. The solution of 300 mg (0.34 mmol) NaBArF in 5 mL dry methanol was added 76 mg（0.30 mmol）**1**·Br. The resulting solution was stirred at room temperature for 12 hours. Then the solvent was removed in vacuo. The residue was suspended in H_2O (5 mL), extracted with CH_2Cl_2 (15 mL × 3). The organic layer was collected, washed with H_2O (5 mL), dried ($MgSO_4$), and concentrated to give **1**·BArF (270 mg, yield 86%). ^{1}H NMR (500 MHz, $CDCl_3$): δ (ppm): 7.69 (s, 8H), 7.56 (s, 4H), 3.04~3.01 (m, 2H), 2.78 (s, 9H), 1.64 (br, 2H), 1.32~1.25 (m, 10H), 0.88 (t, J = 6.5 Hz, 3H); ^{13}C NMR (125 MHz, CD_3OD): δ (ppm): 161.5 (q, $^1J_{CB}$ = 49 Hz), 134.5, 129.1 (q, $^2J_{CF}$ = 33 Hz), 124.4 (q, $^1J_{CF}$ = 270 Hz), 117.1, 66.6, 52.0, 31.4, 28.7, 25.9, 22.5, 22.2, 12.9. HRMS (ESI): $C_{11}H_{26}N^+$ ($\mathbf{1}^+$), calcd *m/z* 172.2060; found *m/z* 172.2079.

2·BArF−**10**·2BArF were prepared from their bromide salts by using similar synthetic method for the preparation of **1**·BArF.

2·BArF. Yield 88%. ^{1}H NMR (500 MHz, $CDCl_3$): δ (ppm): 7.69 (s, 8H), 7.62 (t, *J* = 7.5 Hz, 1H), 7.54 (s, 4H), 7.52 (t, *J* = 7.5 Hz, 2H), 7.23 (d, *J* = 7.5 Hz, 2H), 4.10 (s, 2H), 2.81 (s, 9H). ^{13}C NMR (125 MHz, $CDCl_3$): δ (ppm): 161.7 (q, $^1J_{CB}$ = 50 Hz), 134.7, 132.6, 132.0, 130.3, 129.1 (q, $^2J_{CF}$ = 31 Hz), 124.5 (q, $^1J_{CF}$ = 271 Hz), 124.4, 117.6, 71.8, 53.0. HRMS (ESI): $C_{10}H_{16}N^+$ (**2**$^+$), calcd *m/z* 150.1277; found *m/z* 151.1271.

3·BArF. Yield 83%. ^{1}H NMR (500 MHz, $CDCl_3$): δ (ppm) = 8.13 (d, *J* = 8.5 Hz, 1H), 8.03 (d, *J* = 7.5 Hz, 1H), 7.80 (d, *J* = 8.5 Hz, 1H), 7.70−7.64 (m, 10H), 7.59−7.54 (m, 5H), 7.46 (d, *J* = 7.5 Hz, 1H), 4.68 (s, 2H), 2.86 (s, 9H). ^{13}C NMR (125 MHz, CD_3OD): δ (ppm): 162.5 (q, $^1J_{CB}$ = 50 Hz), 134.4, 134.3, 133.4, 132.9, 131.9, 129.2 (q, $^2J_{CF}$ = 35 Hz), 129.1, 127.6, 127.6 (q, $^1J_{CF}$ = 270 Hz), 126.3, 124.8, 123.7, 122.9, 117.1, 65.1, 52.3. HRMS (ESI): $C_{14}H_{18}N^+$ (**3**$^+$), calcd *m/z* 200.1434; found *m/z* 200.1453.

4·BArF. Yield 78%. ^{1}H NMR (500 MHz, CD_3OD): δ (ppm): 8.47 (d, *J* = 9.5 Hz, 1H), 8.27−8.22 (m, 4H), 8.15−8.12 (m, 2H), 8.06−8.01 (m, 2H), 7.67 (s, 8H), 7.61 (s, 4H), 5.26 (s, 2H), 3.19 (s, 9H). ^{13}C NMR (125 MHz, CD_3OD): δ (ppm): 161.5 (q, $^1J_{CB}$ = 50 Hz), 134.5, 133.3, 131.7, 131.5, 131.1, 130.2, 129.4, 129.1 (q, $^2J_{CF}$ = 35 Hz), 129.0, 126.7, 126.4, 126.3, 125.9, 124.7, 124.4, 124.4 (q, $^1J_{CF}$ = 271 Hz), 124.0, 121.9, 120.2, 117.1, 65.6, 52.1. HRMS (ESI): $C_{20}H_{20}N^+$ (**4**$^+$), calcd *m/z* 274.1590; found *m/z* 274.1587.

5·BArF. Yield 88%. ^{1}H NMR (500 MHz, $CDCl_3$): δ (ppm) = 7.69 (s, 8H), 7.55(s, 4H), 3.08 (t, *J* = 7.5 Hz, 6H), 2.96 (t, *J* = 7.5 Hz, 6H), 2.89−2.85 (m, 2H), 1.33−1.23 (m, 4H), 0.90 (t, *J* = 7.3 Hz, 3H). ^{13}C NMR (125 MHz, CD_2Cl_2): δ (ppm): 159.7 (q, $^1J_{CB}$ = 50 Hz), 132.8,

126.9 (q, $^2J_{CF}$ = 30 Hz), 122.6 (q, $^1J_{CF}$ = 271 Hz), 115.5, 75.2, 63.8, 43.0, 21.8, 17.6, 11.1. HRMS (ESI): $C_{10}H_{21}N_2^+$ (**5**$^+$), calcd *m/z* 169.1699; found *m/z* 169.1695.

6·2BArF. Yield 86%. ^{1}H NMR (500 MHz, CD_2Cl_2): δ (ppm): 7.72 (s, 16H), 7.58 (s, 8H), 3.89 (s, 12H), 3.54−3.50 (m, 4H), 1.79−1.72 (m, 4H), 1.48−1.43 (m, 4H), 1.00 (t, *J* = 7.3 Hz, 6H). ^{13}C NMR (125 MHz, CD_3OD): δ (ppm): 161.5 (q, $^1J_{CB}$ = 50 Hz), 134.4, 129.1 (q, $^2J_{CF}$ = 32 Hz), 124.4 (q, $^1J_{CF}$ = 270 Hz), 117.1, 64.7, 50.9, 23.6, 19.1, 12.3. HRMS (ESI): $C_{46}H_{42}BF_{24}N_2^+$ ([**6**•BArF]$^+$), calcd *m/z* 1089.3052; found *m/z* 1089.3059.

7·2BArF. Yield 85%. ^{1}H NMR (500 MHz, CD_2Cl_2): δ (ppm): 8.78 (d, *J* = 5.0 Hz , 4H), 8.20 (d, *J* = 4.5 Hz, 4H), 7.75 (s, 16H), 7.59 (s, 8H), 4.67 (t, *J* = 7.5 Hz, 2H), 2.12−2.05 (m, 4H), 1.51−1.46 (m, 4H), 1.05 (t, *J* = 7.5 Hz, 6H). ^{13}C NMR (125 MHz, CD_3OD): δ (ppm): 161.5 (q, $^1J_{CB}$ = 50 Hz), 150, 145.7, 134.4, 129.1 (q, $^2J_{CF}$ = 32 Hz), 126.8, 124.4 (q, $^1J_{CF}$ =270 Hz), 117.1, 61.7, 33.1, 19.0, 12.3. HRMS (ESI): $C_{50}H_{38}BF_{24}N_2^+$ ([**7**•BArF]$^+$), calcd m/z 1133.2739; found m/z 1133.30.

8·2BArF. Yield 85%. ^{1}H NMR (500 MHz, CD_2Cl_2): δ (ppm): 8.46 (d, *J* = 6.5 Hz, 4H), 7.98 (d, *J* = 6.5 Hz, 4H), 7.72 (s, 16H), 7.55 (s, 8H), 7.50 (s, 2H), 4.48 (t, *J* = 7.5 Hz, 4H), 2.02−1.96 (m, 4H), 1.42−1.36 (m, 4H), 0.98 (t, *J* = 7.3 Hz, 6H). ^{13}C NMR (125 MHz, CD_2Cl_2): δ (ppm): 161.7 (q, $^1J_{CB}$ = 50 Hz), 150.9, 143.9, 134.8, 133.6, 128.9 (q, $^2J_{CF}$ = 34 Hz), 126.4, 124.6 (q, $^1J_{CF}$ = 271 Hz), 117.5, 62.9, 33.2, 19.3, 12.8. HRMS (ESI): $C_{52}H_{40}BF_{24}N_2^+$ ([**8**•BArF]$^+$) calcd m/z 1159.2896; found m/z 1159.2905.

9·2BArF. Yield 79%. ^{1}H NMR (500 MHz, CD_2Cl_2): δ (ppm): 9.01−8.98 (m, 4H), 8.75 (d, *J* = 8.0 Hz, 2H), 8.51 (t, *J* = 7.0 Hz, 2H), 7.75 (s, 16H), 7.59 (s, 8H), 5.32 (s, 4H). ^{13}C NMR (125 MHz, CD_3OD): δ (ppm): 161.5 (q, $^1J_{CB}$ = 50 Hz), 148.0, 147.2, 140.2, 134.4, 130.4,

129.1 (q, $^2J_{CF}$ = 32 Hz), 128.0, 124.4 (q, $^1J_{CF}$ = 270 Hz), 117.1, 52.4. HRMS (ESI): $C_{44}H_{24}BF_{24}N_2^+$ ([**9**•BArF]$^+$) calcd m/z 1047.1644; found m/z 1047.1656.

10·2BArF. Yield 81%. ^{1}H NMR (500 MHz, CD_2Cl_2): δ (ppm): 9.48 (d, *J* = 8.5 Hz 2H), 9.40 (d, *J* = 5.5 Hz 2H), 8.72−8.68 (m, 4H), 7.74 (s, 16H), 7.57 (s, 8H), 5.69 (s, 4H). ^{13}C NMR (125 MHz, CD_3OD): δ (ppm): 161.5 (q, $^1J_{CB}$ = 50 Hz), 149, 147.9, 134.4, 131.8, 129.7, 129.1 (q, $^2J_{CF}$ = 32 Hz), 127.5, 124.4 (q, $^1J_{CF}$ = 270 Hz), 117.1, 52.3. HRMS (ESI): $C_{46}H_{24}BF_{24}N_2^+$ ([**10**•BArF]$^+$), calcd *m/z* 1071.1644; found *m/z* 1071.1649.

Crystal data of EtBP3 and EtBP4.

Table S1. Crystal data and structure refinement for EtBP3 (CCDC 1016929).

Identification code	mo_30725a	
Empirical formula	C51 H54 O6	
Formula weight	762.94	
Temperature	193(2) K	
Wavelength	0.71073 Å	
Crystal system	Triclinic	
Space group	P -1	
Unit cell dimensions	a = 13.7149(12) Å	α= 103.4550(10)°.
	b = 15.9855(13) Å	β= 105.8790(10)°.
	c = 20.8687(17) Å	γ = 93.251(2)°.
Volume	4244.3(6) $Å^3$	
Z	4	
Density (calculated)	1.194 Mg/m^3	
Absorption coefficient	0.077 mm^{-1}	
F(000)	1632	
Crystal size	0.110 x 0.080 x 0.070 mm^3	
Theta range for data collection	1.050 to 25.009°.	
Index ranges	-16<=h<=15, -13<=k<=19, -24<=l<=24	
Reflections collected	25906	
Independent reflections	14799 [R(int) = 0.0331]	
Completeness to theta = 25.242°	96.4 %	
Absorption correction	Semi-empirical from equivalents	
Max. and min. transmission	0.746 and 0.694	
Refinement method	Full-matrix least-squares on F^2	
Data / restraints / parameters	14799 / 4 / 1053	
Goodness-of-fit on F^2	0.972	
Final R indices [I>2sigma(I)]	R1 = 0.0557, wR2 = 0.1635	
R indices (all data)	R1 = 0.1063, wR2 = 0.2363	
Extinction coefficient	n/a	
Largest diff. peak and hole	0.452 and -0.430 e.$Å^{-3}$	

Table S2. Crystal data and structure refinement for EtBP4 (CCDC 1016930).

Identification code	mo_31101aa	
Empirical formula	C68 H72 O8	
Formula weight	1017.25	
Temperature	173(2) K	
Wavelength	0.71073 Å	
Crystal system	Monoclinic	
Space group	P 21/c	
Unit cell dimensions	a = 11.5532(19) Å	α= 90°.
	b = 30.664(5) Å	β= 90.771(3)°.
	c = 7.7100(13) Å	γ = 90°.
Volume	2731.1(8) Å^3	
Z	2	
Density (calculated)	1.237 Mg/m^3	
Absorption coefficient	0.080 mm^{-1}	
F(000)	1088	
Crystal size	0.280 x 0.110 x 0.080 mm^3	
Theta range for data collection	1.763 to 26.006°.	
Index ranges	-14<=h<=14, -36<=k<=37, -9<=l<=9	
Reflections collected	17628	
Independent reflections	5369 [R(int) = 0.0454]	
Completeness to theta = 25.242°	99.8 %	
Absorption correction	Semi-empirical from equivalents	
Refinement method	Full-matrix least-squares on F^2	
Data / restraints / parameters	5369 / 0 / 347	
Goodness-of-fit on F^2	1.068	
Final R indices [I>2sigma(I)]	R1 = 0.0550, wR2 = 0.1501	
R indices (all data)	R1 = 0.0795, wR2 = 0.1635	
Extinction coefficient	n/a	
Largest diff. peak and hole	0.361 and -0.244 e.Å^{-3}	

Copies of ^{1}H NMR and ^{13}C NMR spectra of hosts and guests.

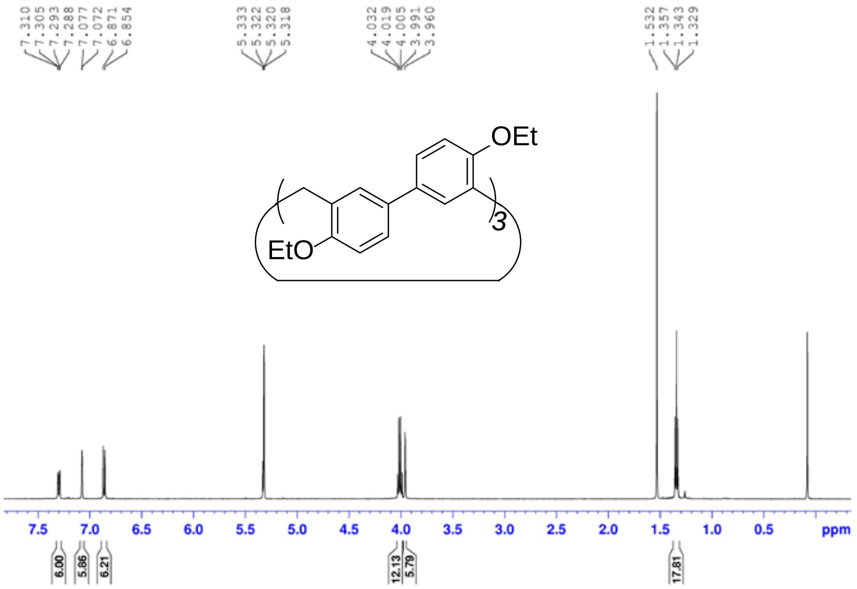

Figure S1. ^{1}H NMR spectrum (500 MHz) of EtBP3 in CD_2Cl_2.

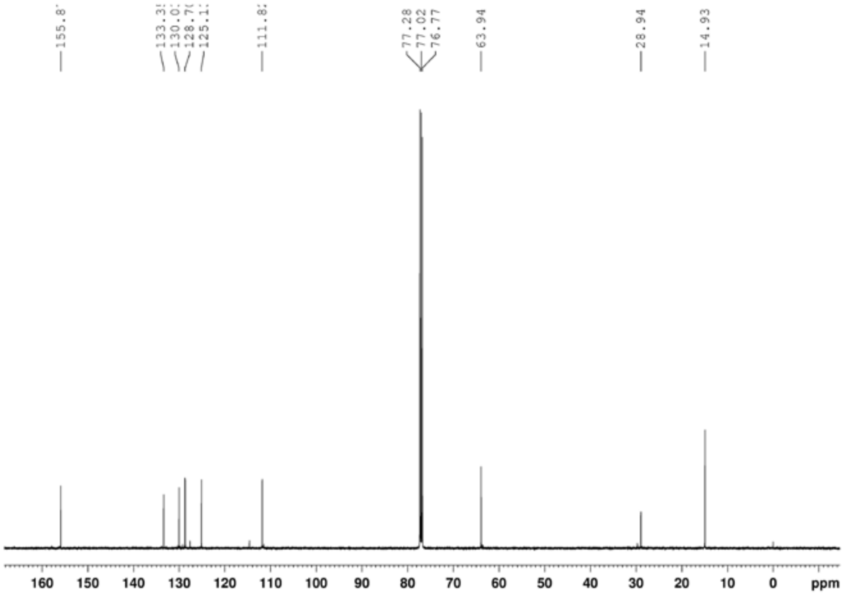

Figure S2. ^{13}C NMR spectrum (125 MHz) of EtBP3 in $CDCl_3$.

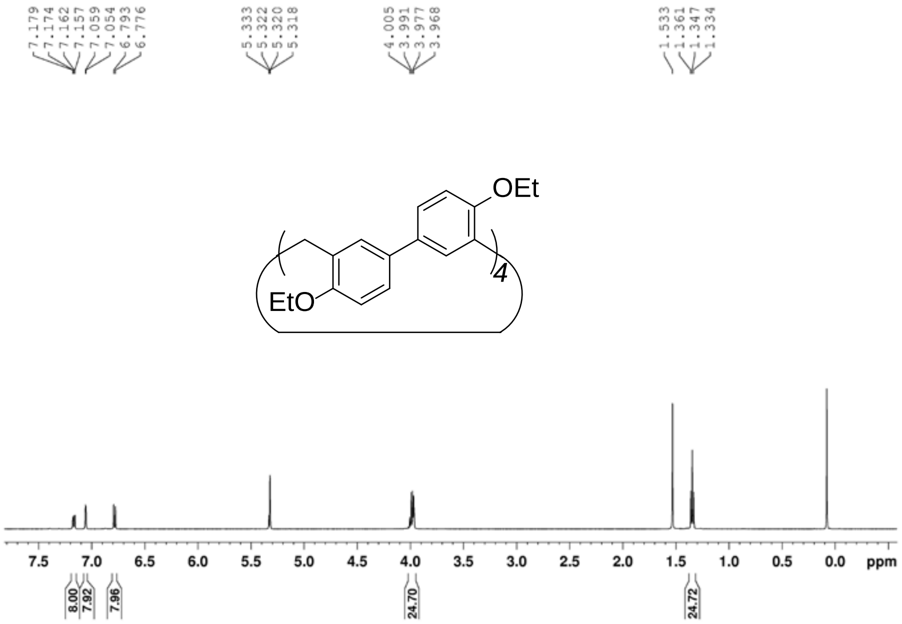

Figure S3. ^{1}H NMR spectrum (500 MHz) of EtBP4 in CD_2Cl_2.

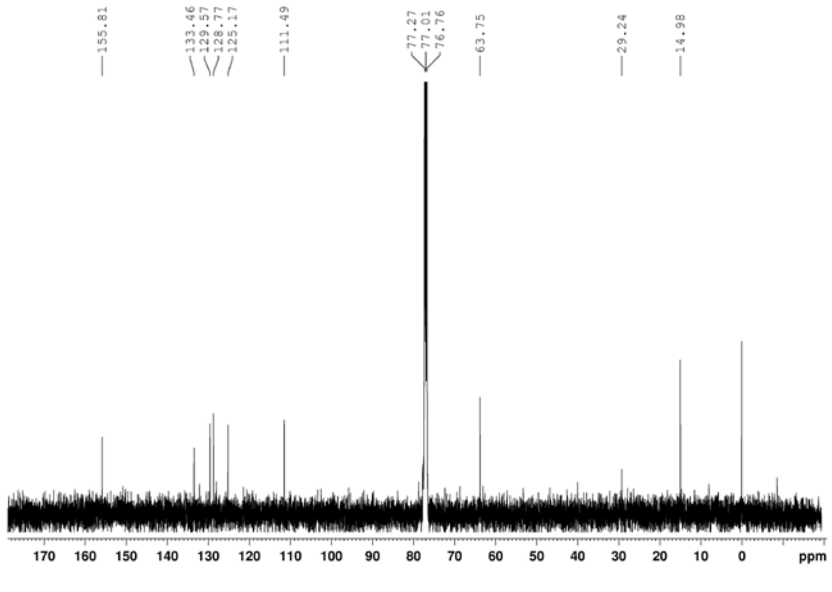

Figure S4. ^{13}C NMR spectrum (125 MHz) of EtBP4 in $CDCl_3$.

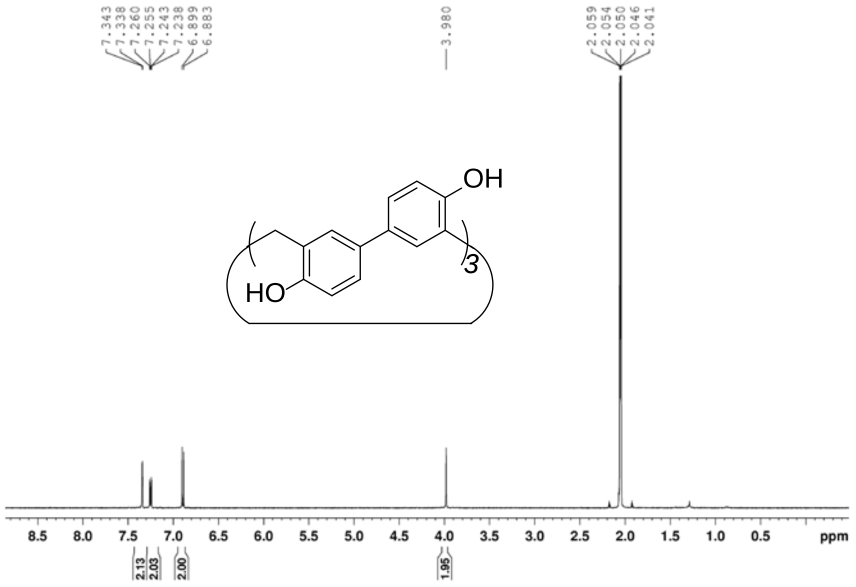

Figure S5. ^{1}H NMR spectrum (500 MHz) of OHBP3 in $(CD_3)_2CO$.

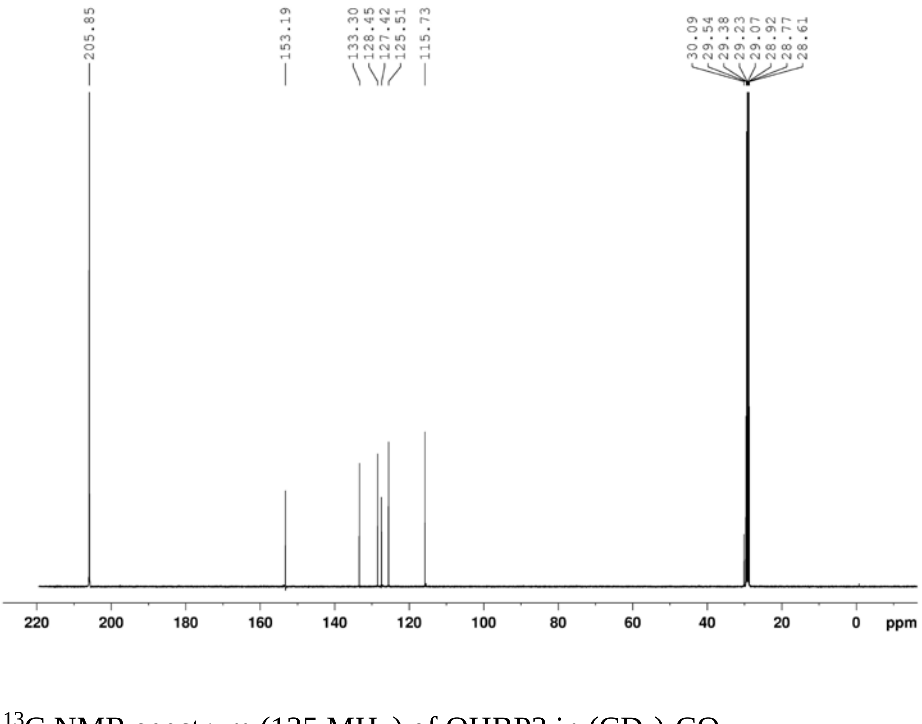

Figure S6. ^{13}C NMR spectrum (125 MHz) of OHBP3 in $(CD_3)_2CO$.

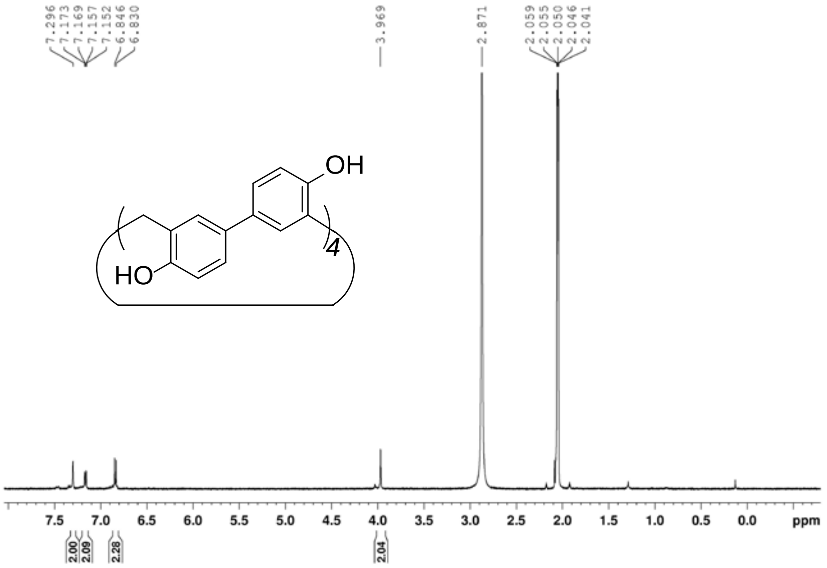

Figure S7. ^{1}H NMR spectrum (500 MHz) of OHBP4 in $(CD_3)_2CO$.

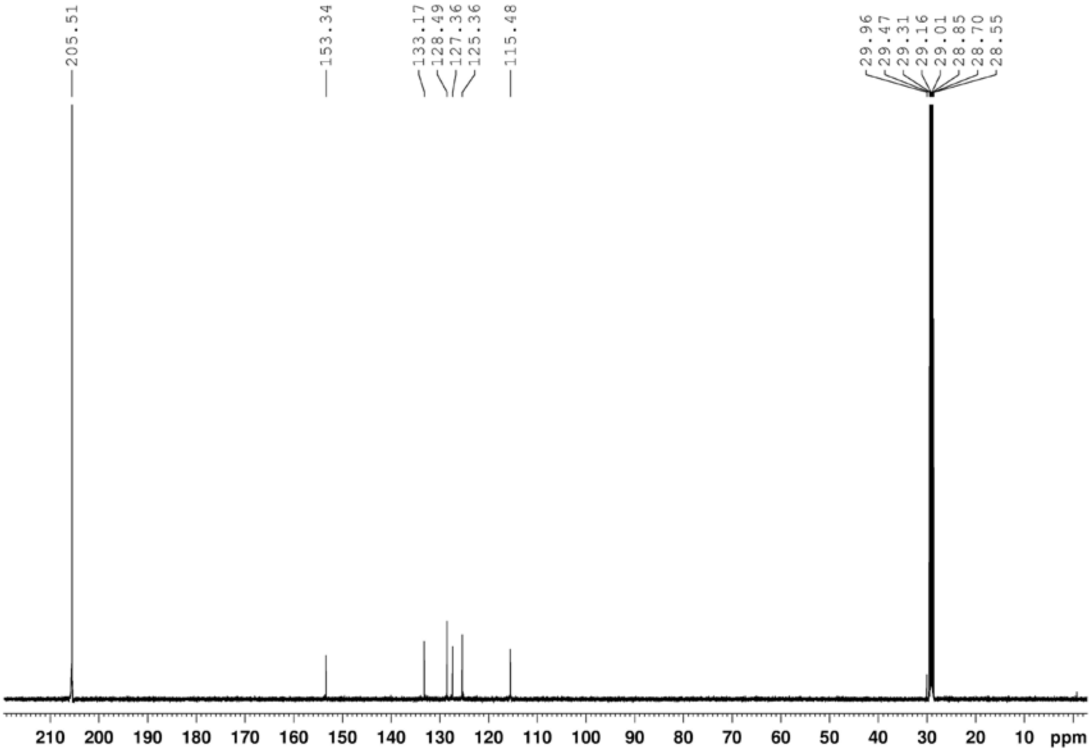

Figure S8. ^{13}C NMR spectrum (125 MHz) of OHBP4 in $(CD_3)_2CO$.

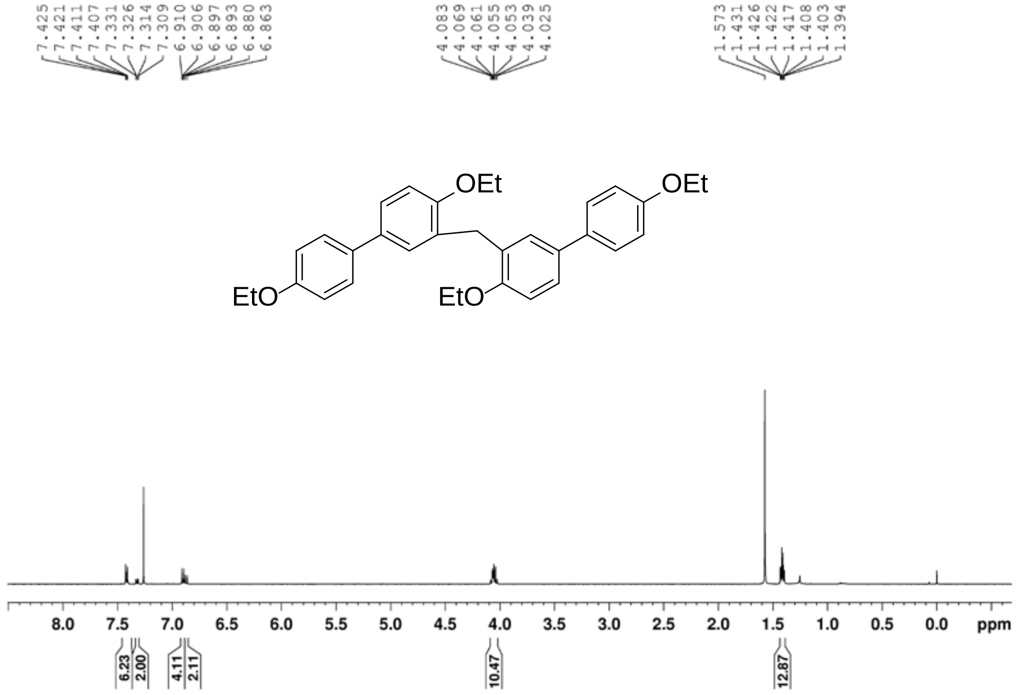

Figure S9. ^{1}H NMR spectrum (500 MHz) of BPD (the acyclic dimer) in $CDCl_3$.

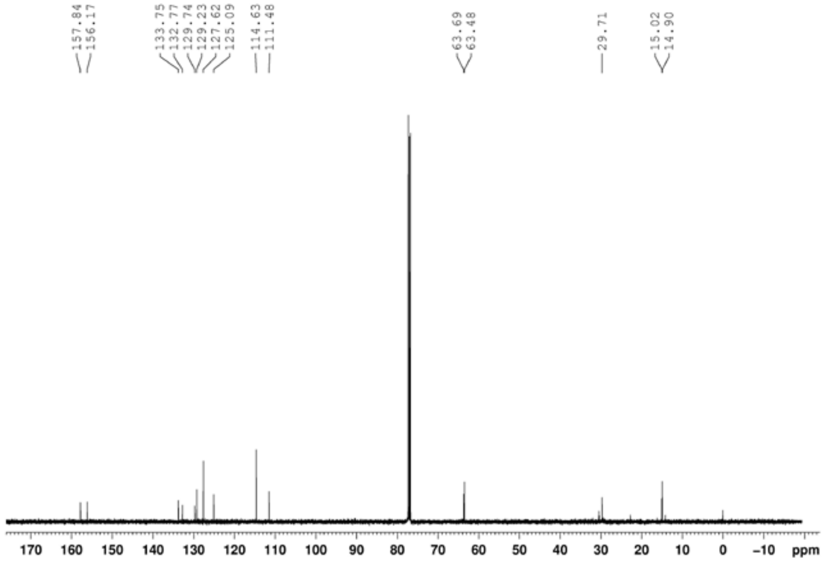

Figure S10. ^{13}C NMR spectrum (125 MHz) of BPD (the acyclic dimer) in $CDCl_3$.

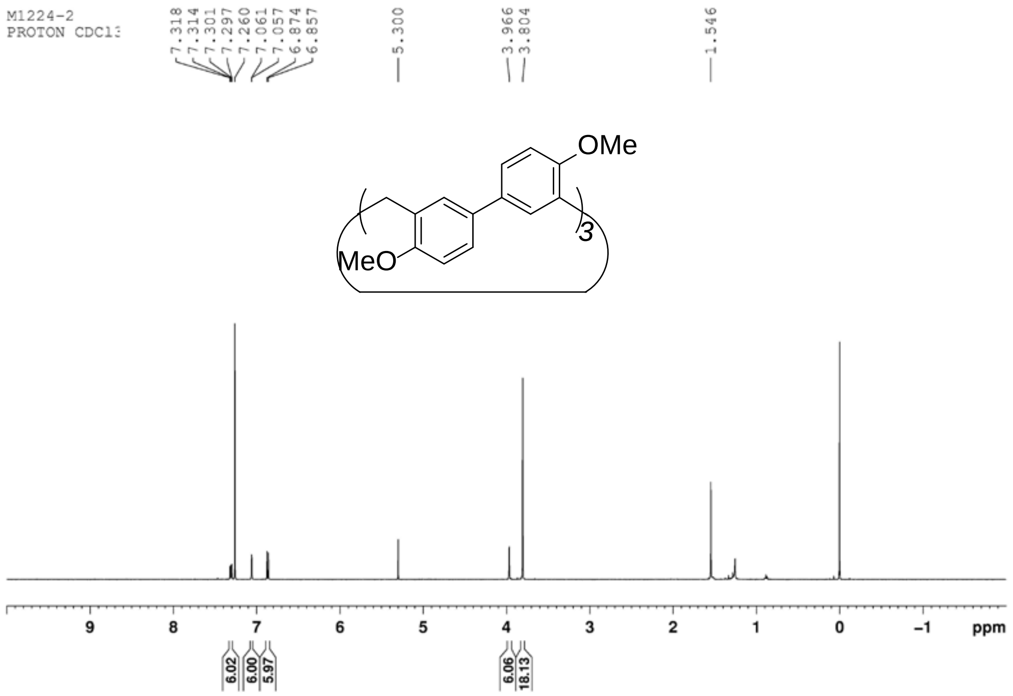

Figure S11. ^{1}H NMR spectrum (500 MHz) of MeBP3 in $CDCl_3$.

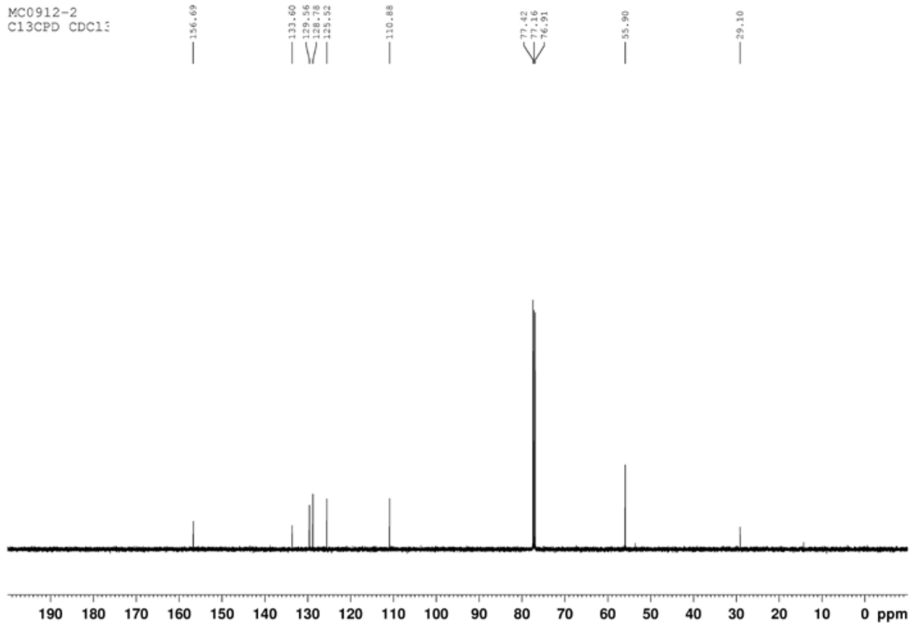

Figure S12. ^{13}C NMR spectrum (125 MHz) of MeBP3 in $CDCl_3$.

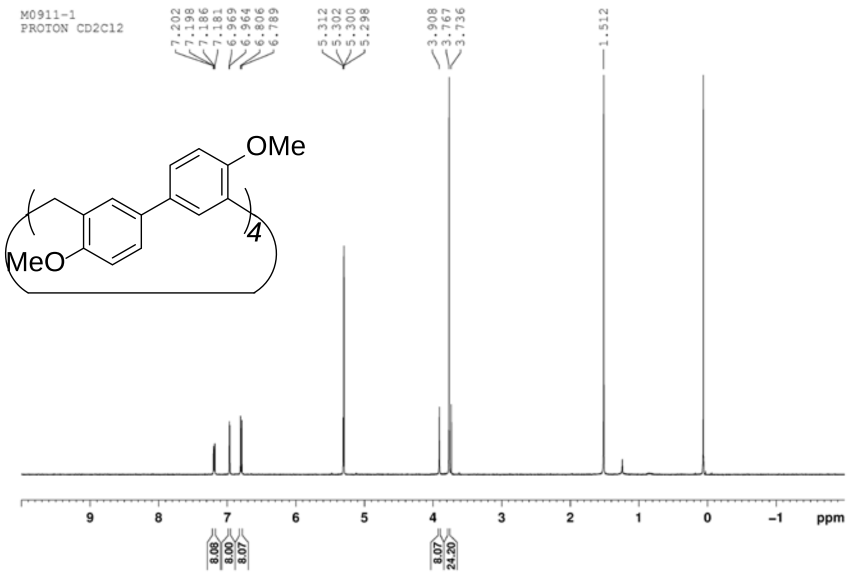

Figure S13. ^{1}H NMR spectrum (500 MHz) of MeBP4 in CD_2Cl_2. The peak at 3.74 ppm is the proton signal of 1,2-dichloroethane.

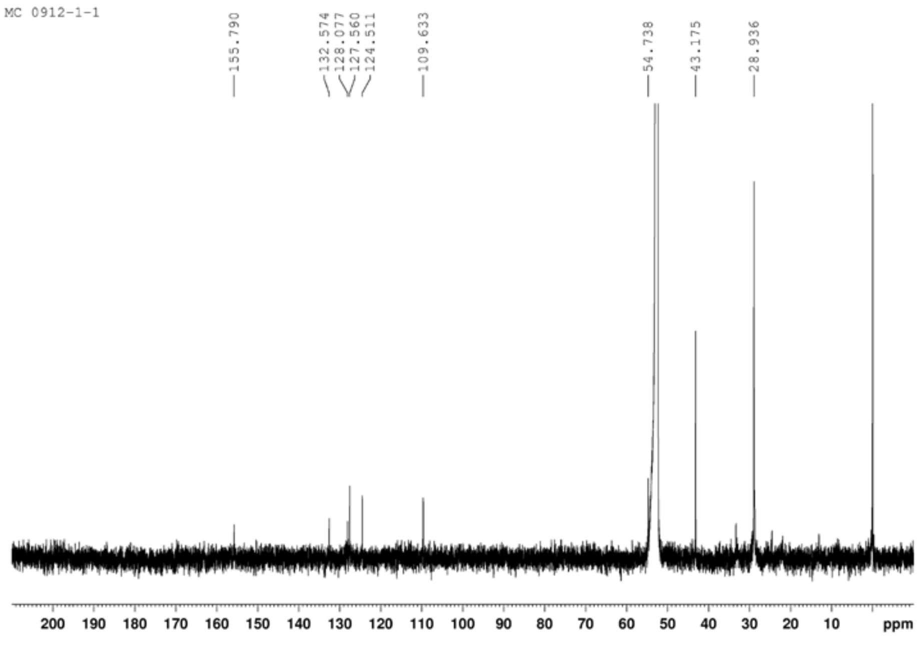

Figure S14. ^{13}C NMR spectrum (175 MHz) of MeBP4 in CD_2Cl_2. The peak at 43.2 ppm is the carbon signal of 1,2-dichloroethane.

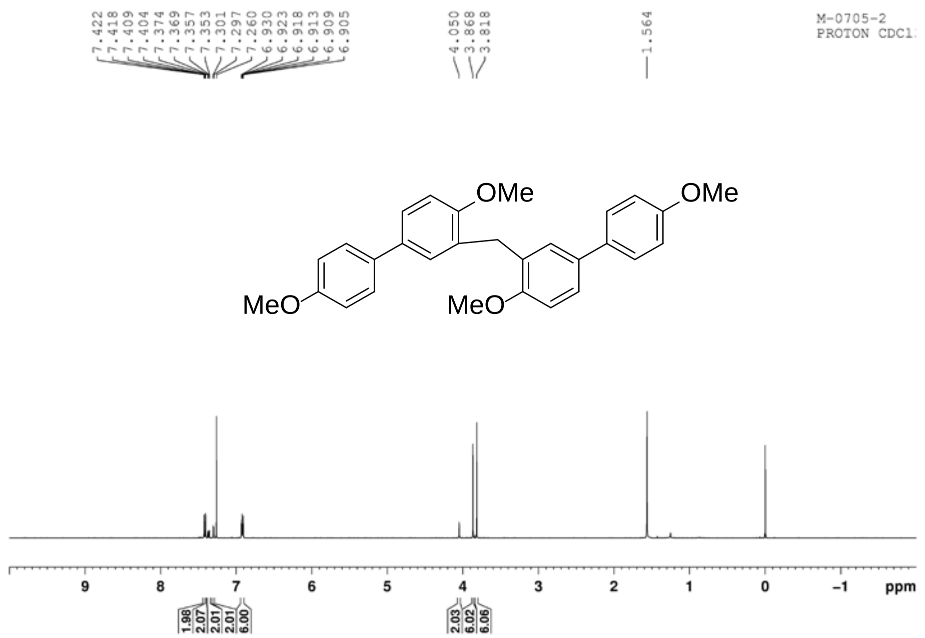

Figure S15. ^{1}H NMR spectrum (500 MHz) of the acyclic dimer in $CDCl_3$.

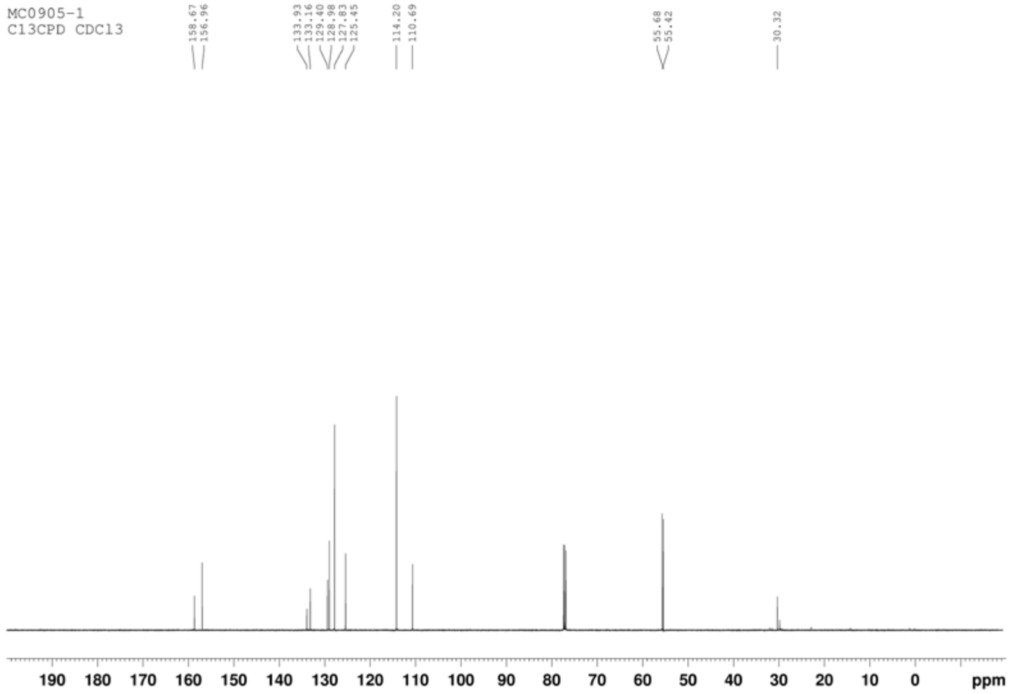

Figure S16. ^{13}C NMR spectrum (125 MHz) of the acyclic dimer in $CDCl_3$.

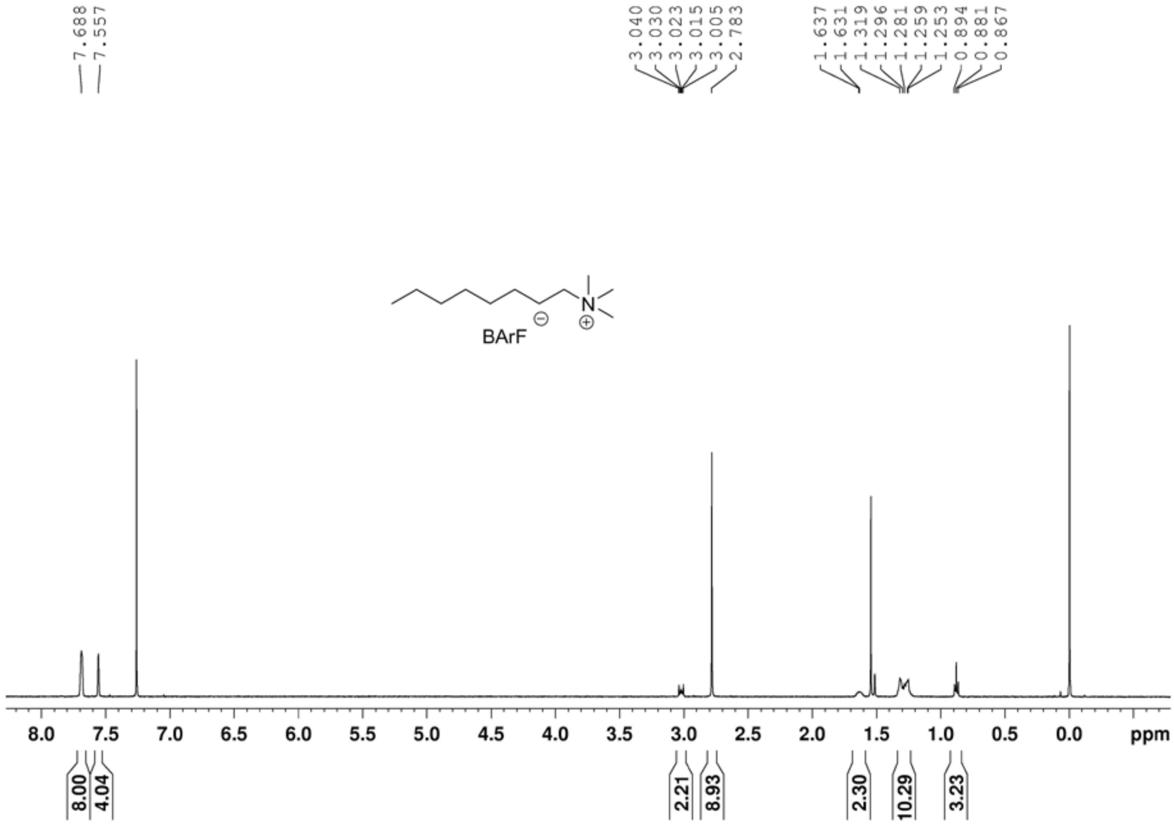

Figure S17. ^{1}H NMR spectrum (500 MHz) of **1**·BArF in $CDCl_3$.

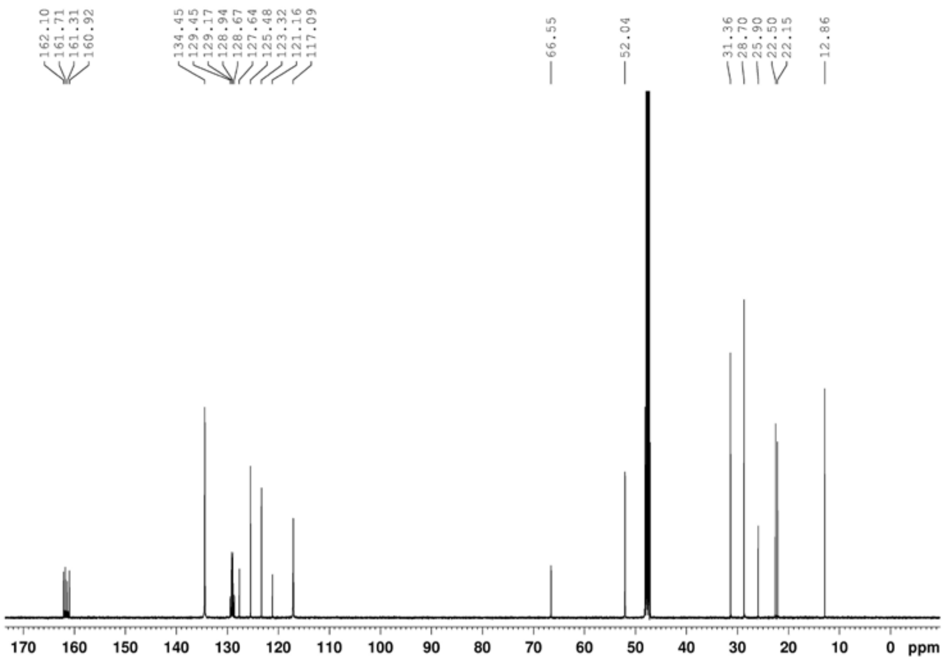

Figure S18. ^{13}C NMR spectrum (125 MHz) of **1**·BArF in CD_3OD.

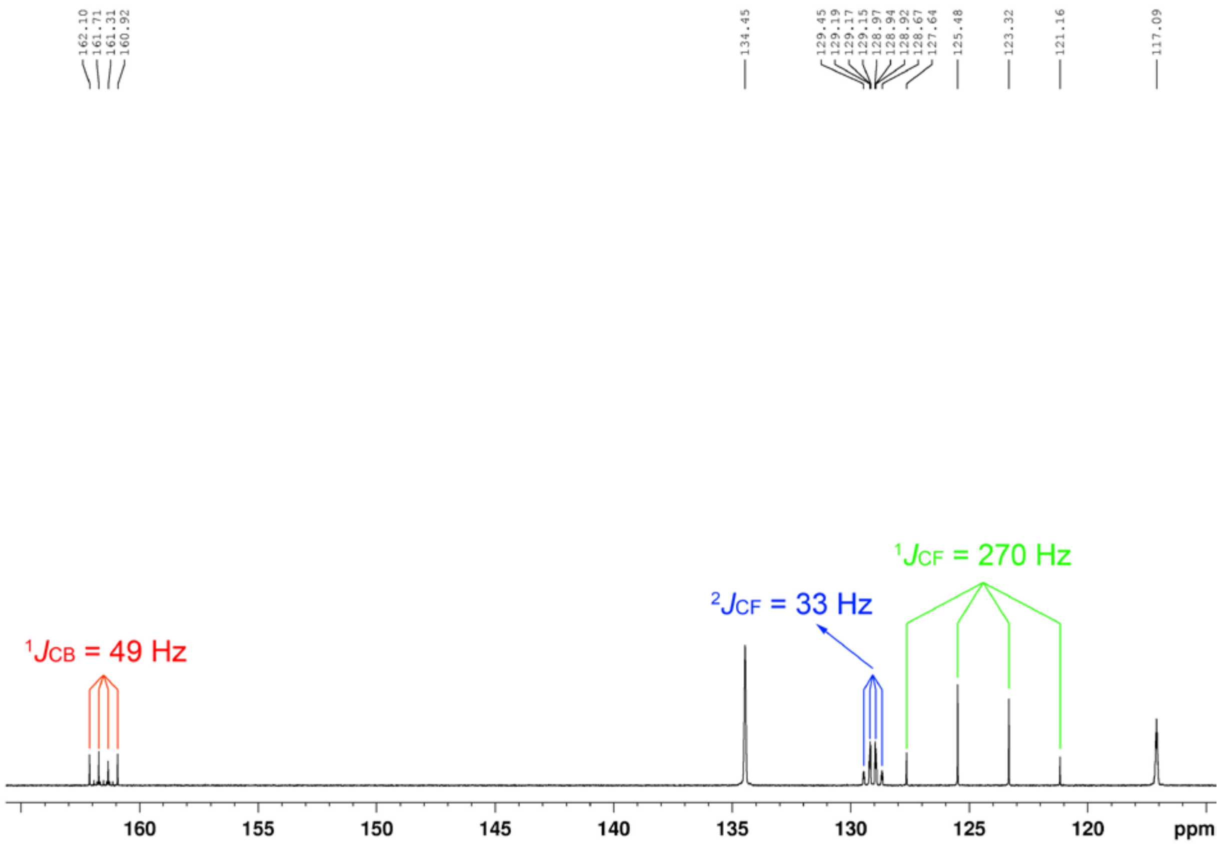

Figure S19. Partial ^{13}C NMR spectrum (125 MHz) of **1**·BArF in CD_3OD.

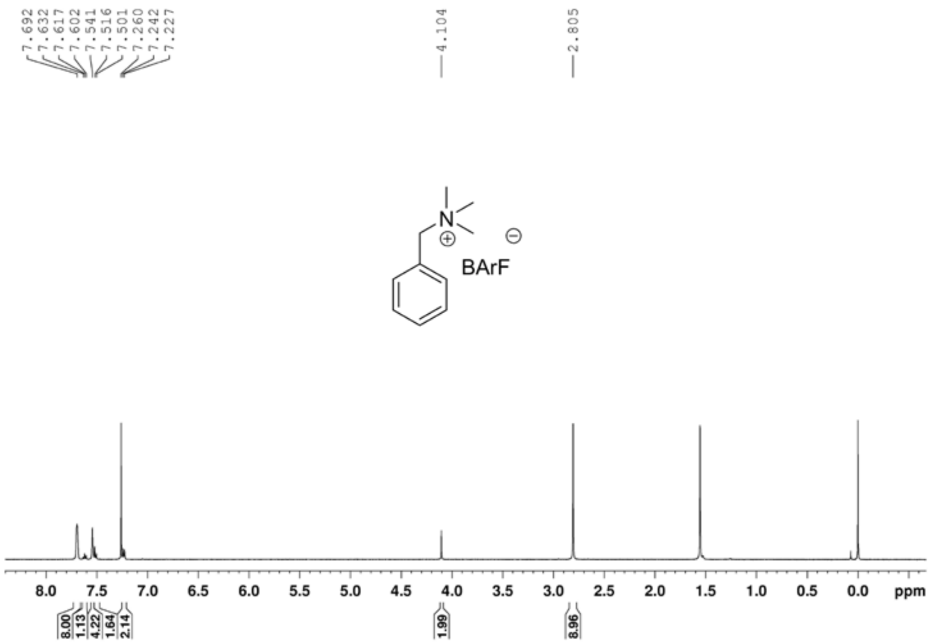

Figure S20. ^{1}H NMR spectrum (500 MHz) of **2**·BArF in $CDCl_3$.

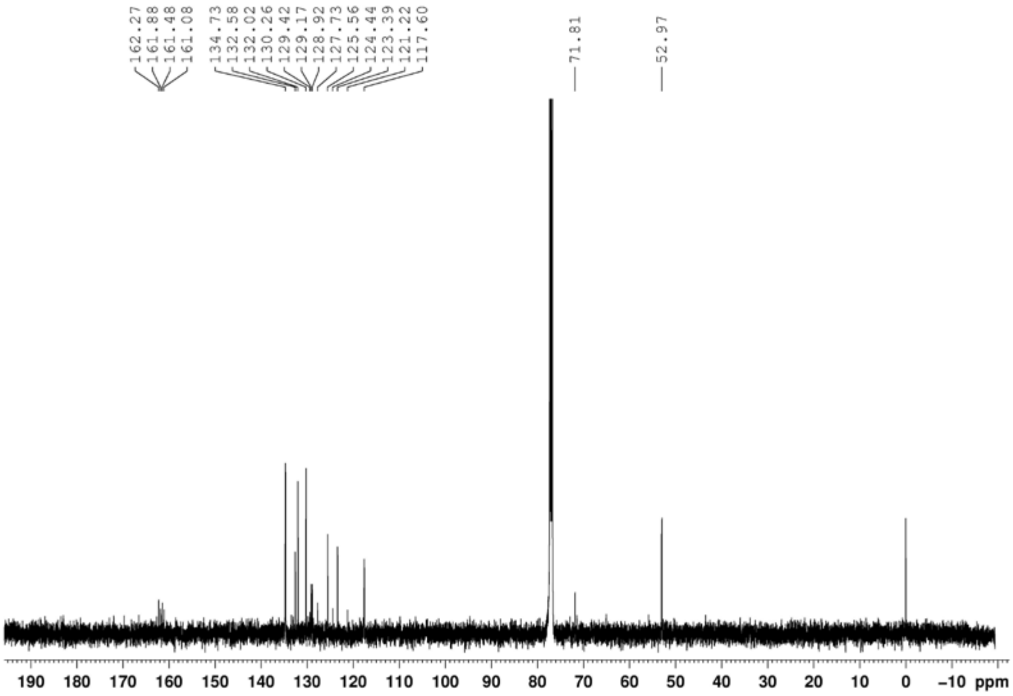

Figure S21. ^{13}C NMR spectrum (125 MHz) of **2**·BArF in $CDCl_3$.

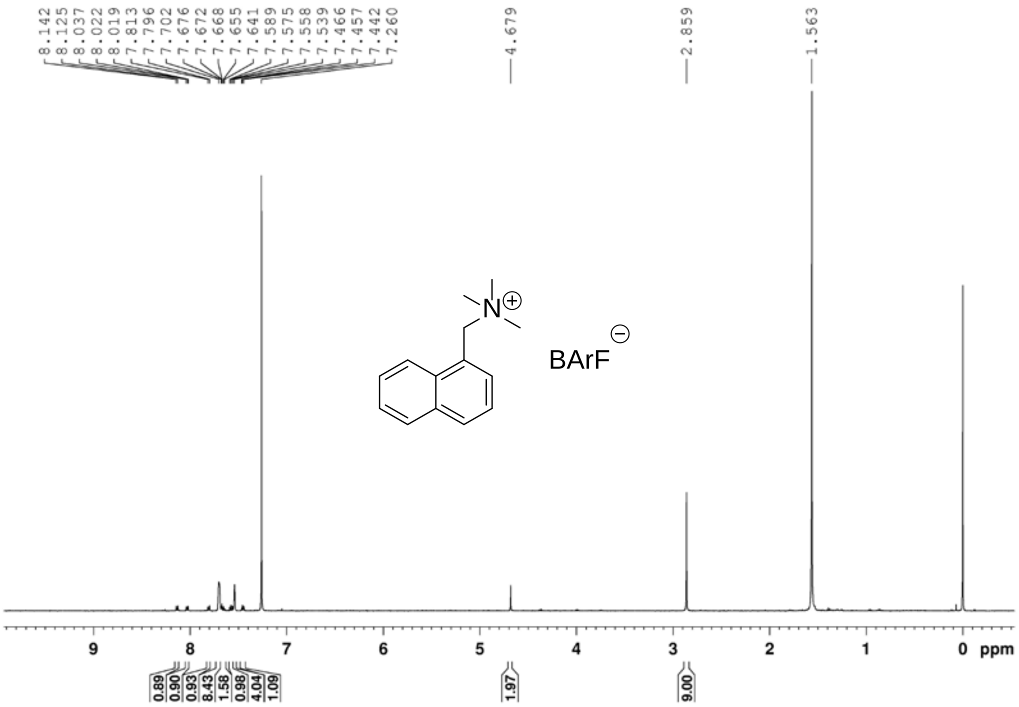

Figure S22. ^{1}H NMR spectrum (500 MHz) of **3**·BArF in $CDCl_3$.

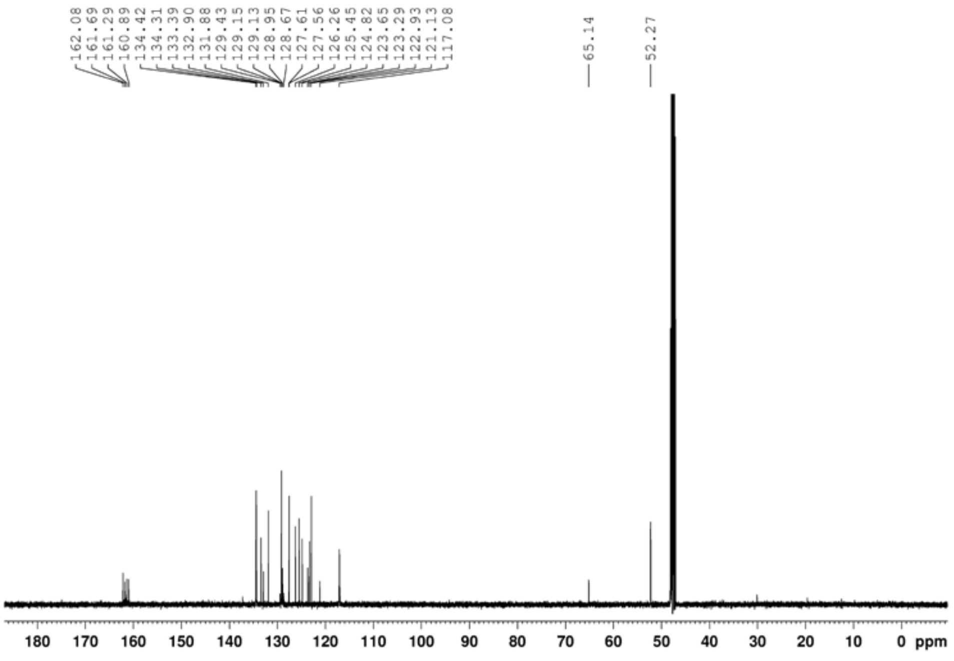

Figure S23. ^{13}C NMR spectrum (125 MHz) of **3**·BArF in CD_3OD.

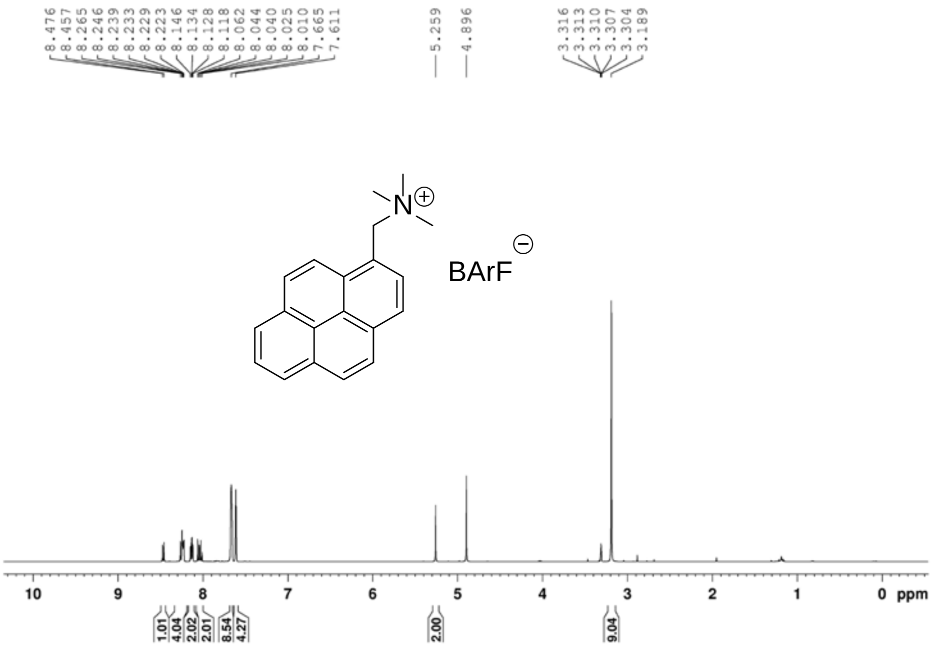

Figure S24. ^{1}H NMR spectrum (500 MHz) of **4**·BArF in CD_3OD.

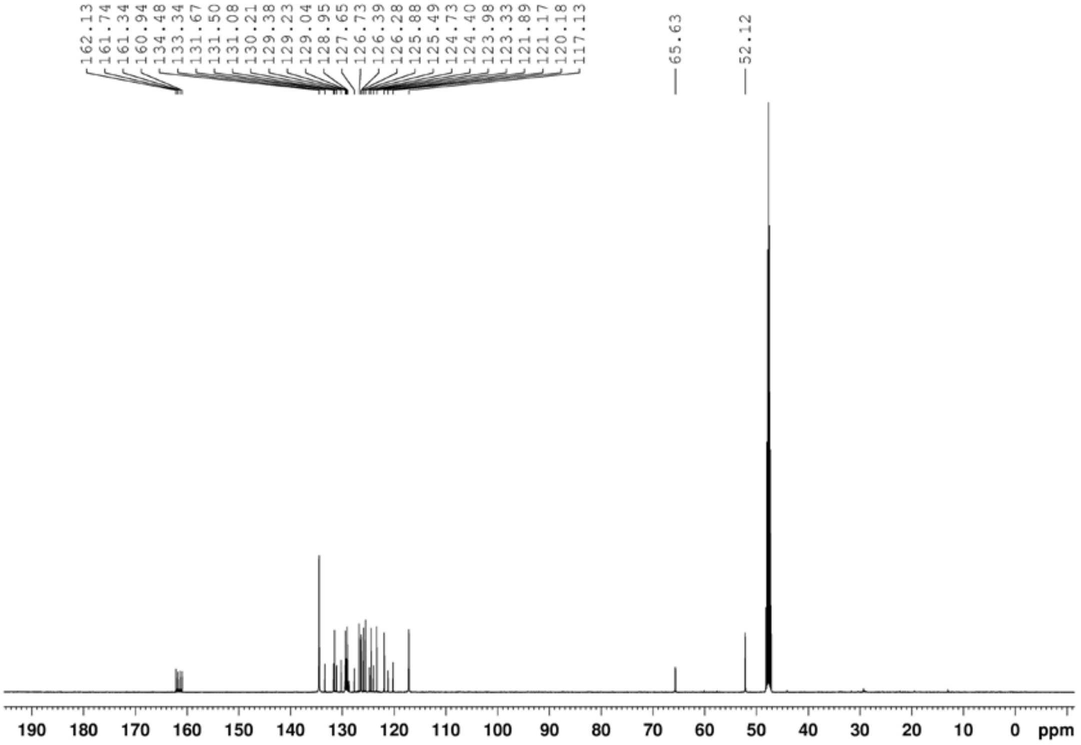

Figure S25. ^{13}C NMR spectrum (125 MHz) of **4**·BArF in CD_3OD.

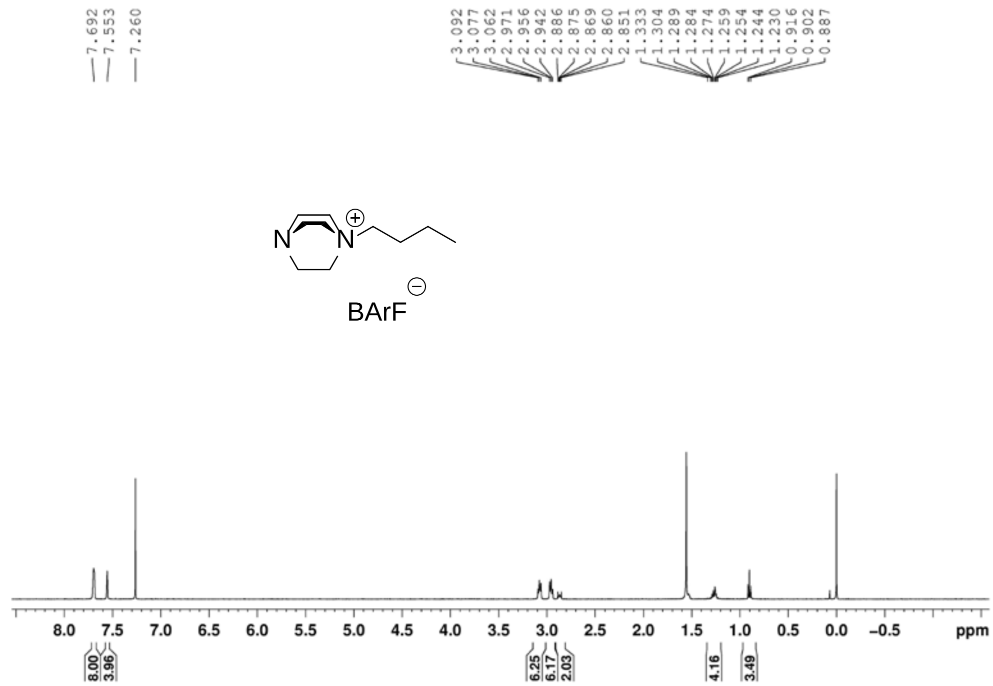

Figure S26. ^{1}H NMR spectrum (500 MHz) of **5**·BArF in $CDCl_3$.

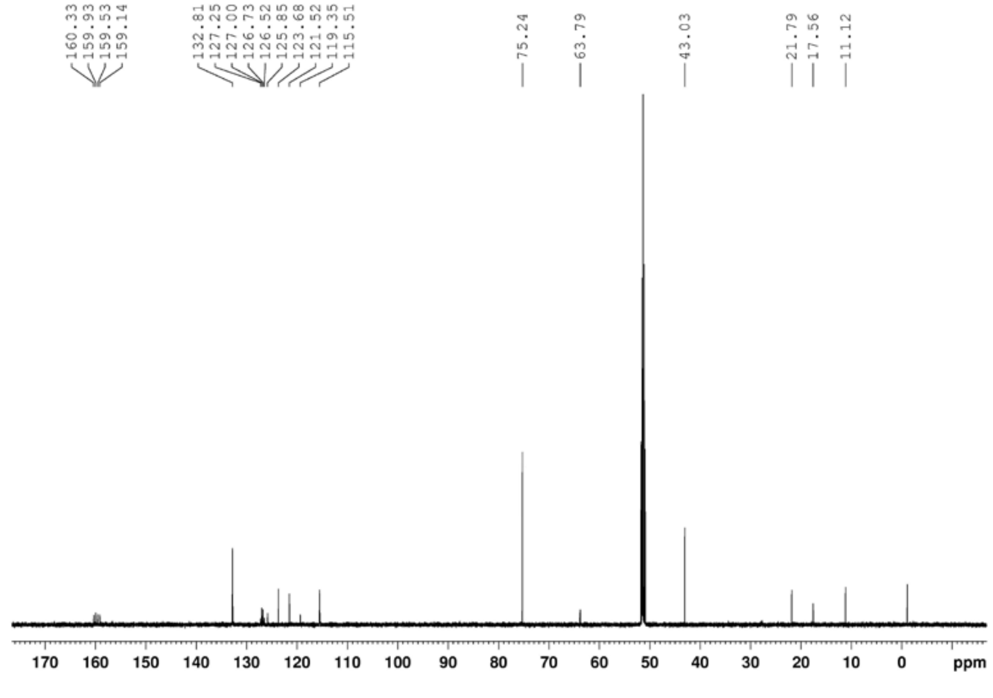

Figure S27. ^{13}C NMR spectrum (125 MHz) of **5**·BArF in CD_2Cl_2.

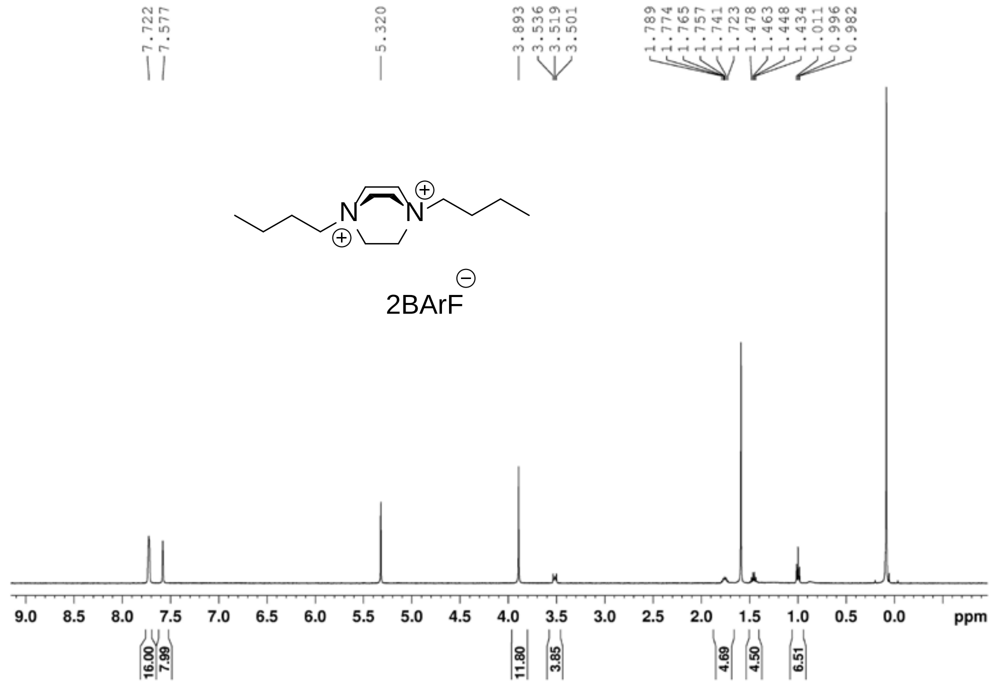

Figure S28. ^{1}H NMR spectrum (500 MHz) of **6**·2BArF in CD_2Cl_2.

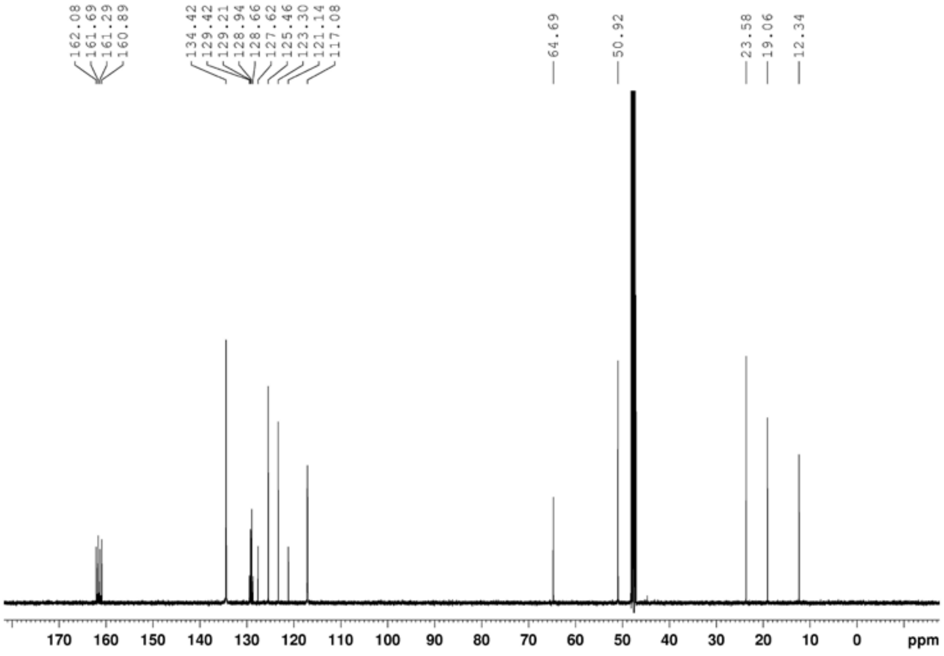

Figure S29. ^{13}C NMR spectrum (125 MHz) of **6**·2BArF in CD_3OD.

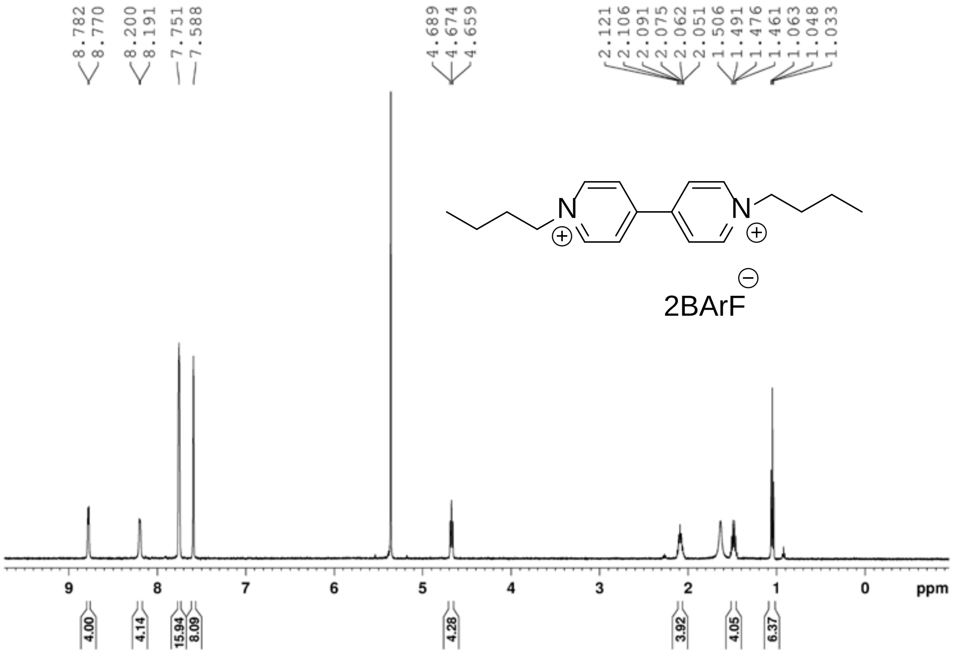

Figure S30. ^{1}H NMR spectrum (500 MHz) of **7**·2BArF in CD_2Cl_2.

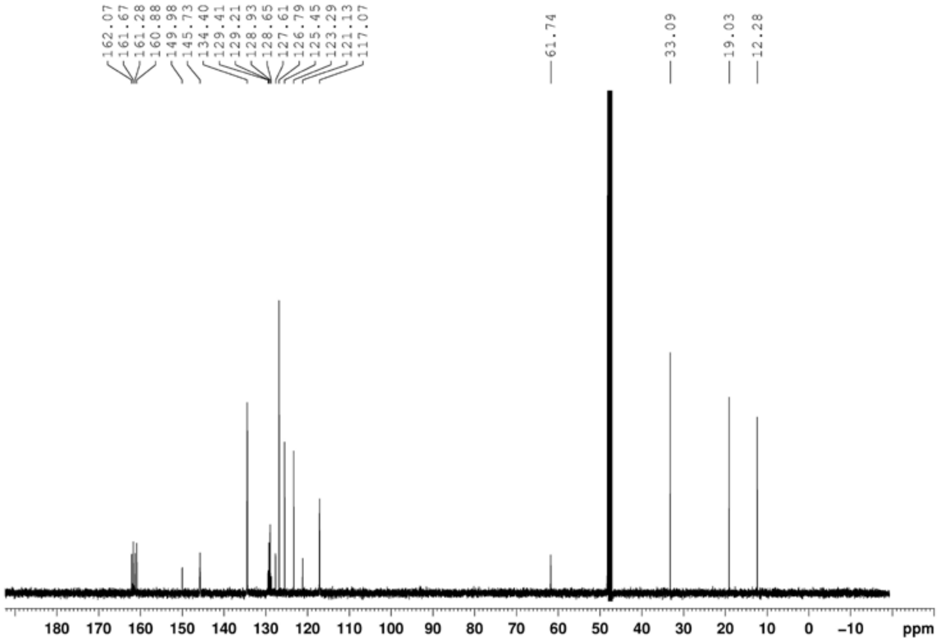

Figure S31. ^{13}C NMR spectrum (125 MHz) of **7**·2BArF in CD_3OD.

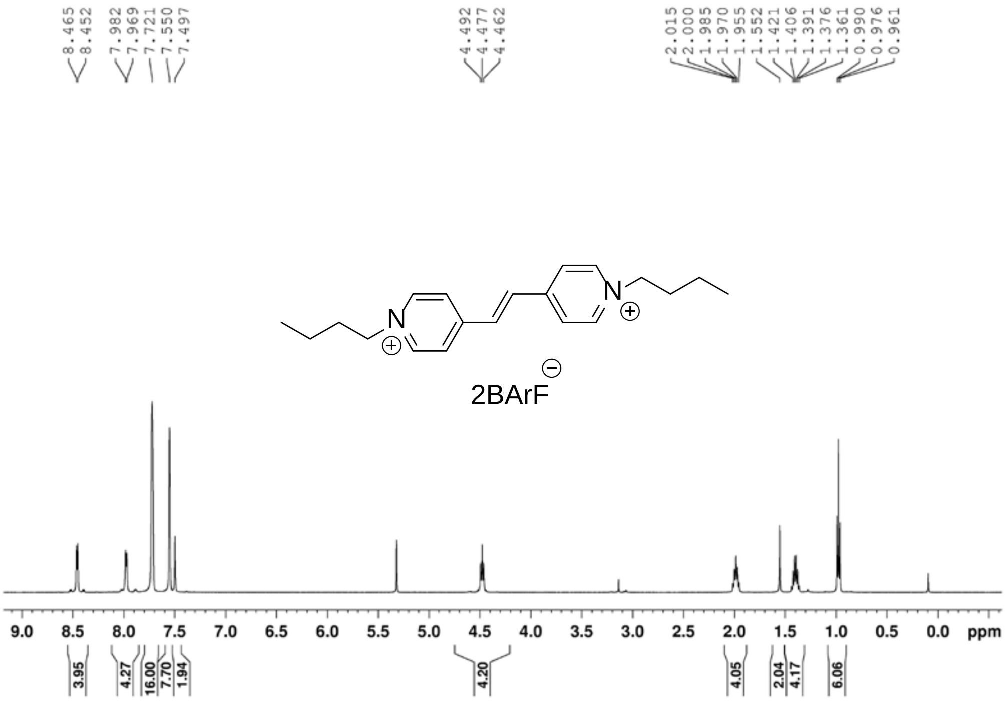

Figure S32. ^{1}H NMR spectrum (500 MHz) of **8**·2BArF in CD_2Cl_2.

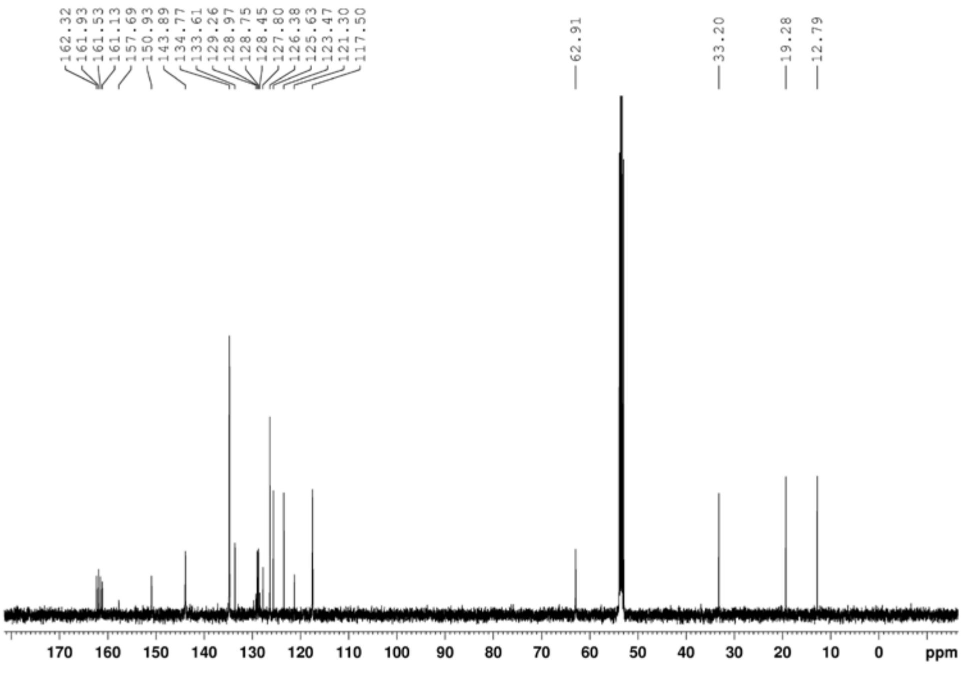

Figure S33. ^{13}C NMR spectrum (125 MHz) of **8**·2BArF in CD_2Cl_2.

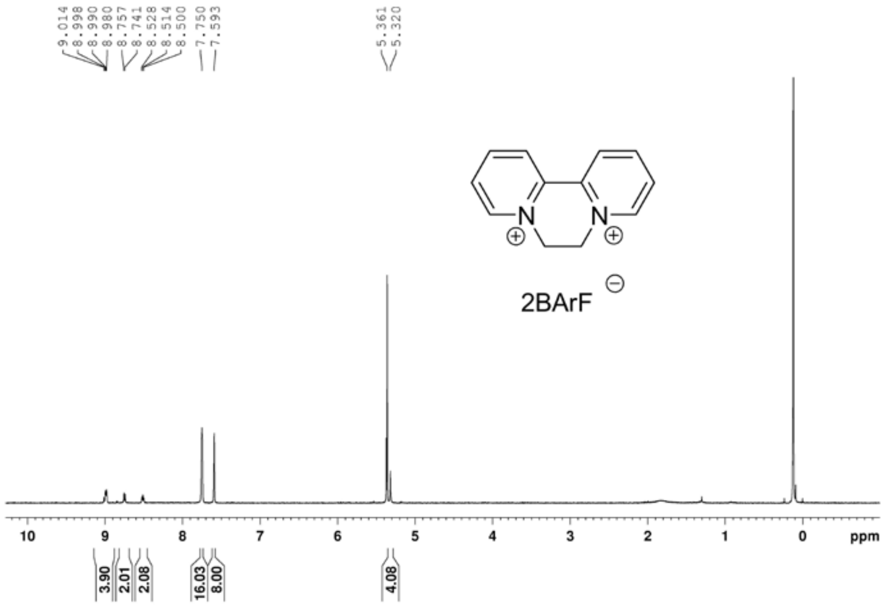

Figure S34. ^{1}H NMR spectrum (500 MHz) of **9**·2BArF in CD_2Cl_2.

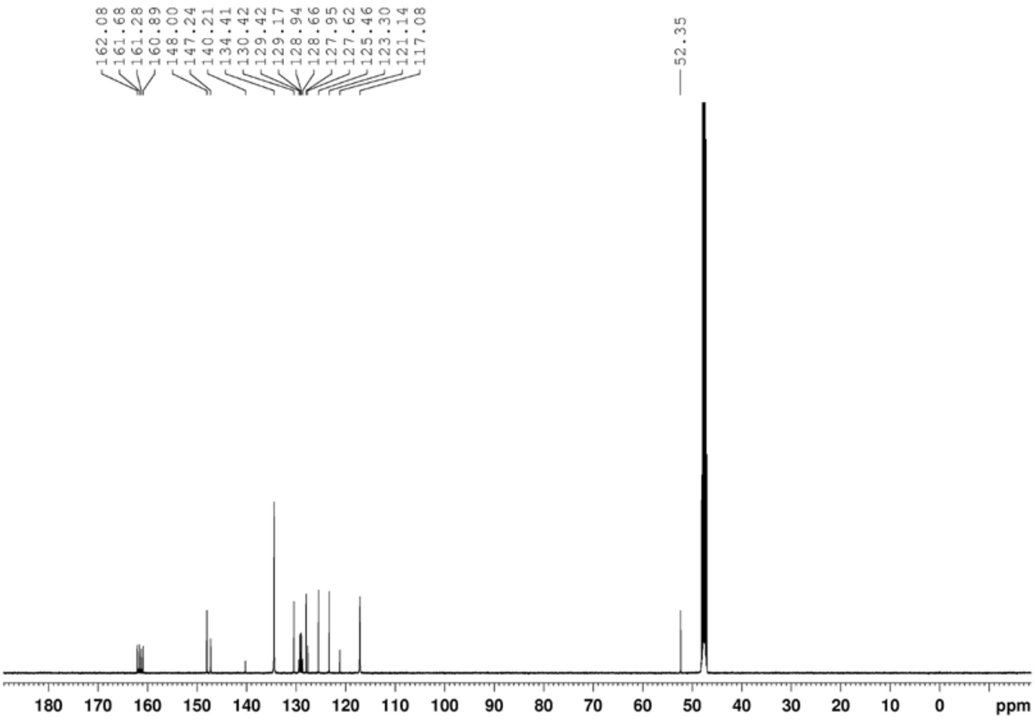

Figure S35. ^{13}C NMR spectrum (125 MHz) of **9**·2BArF in CD_3OD.

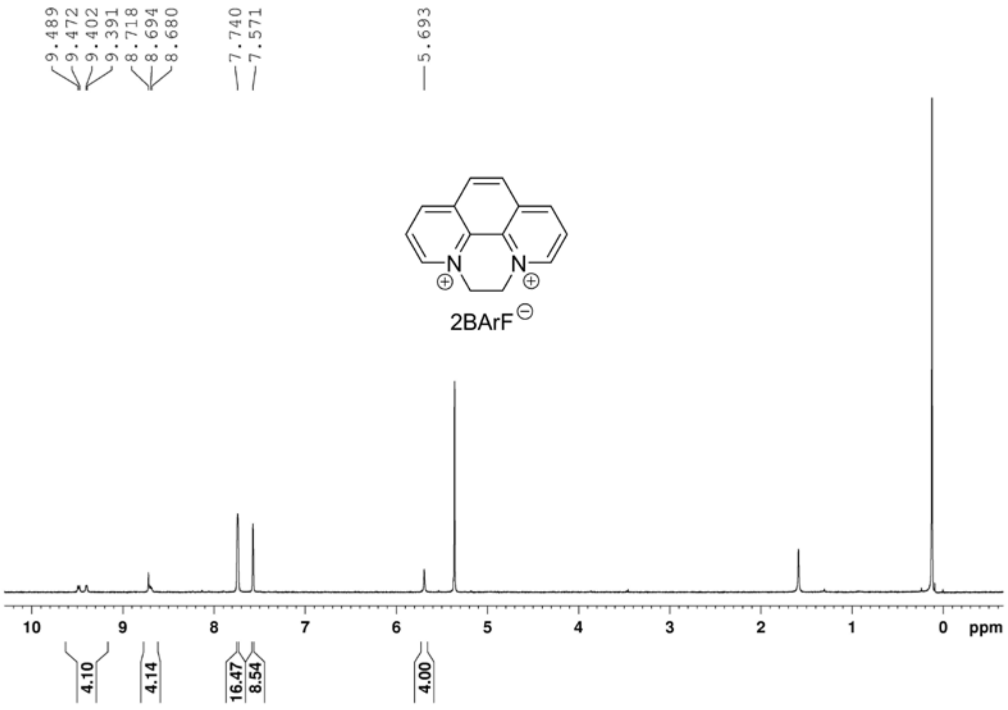

Figure S36. ^{1}H NMR spectrum (500 MHz) of **10**·2BArF in CD_2Cl_2.

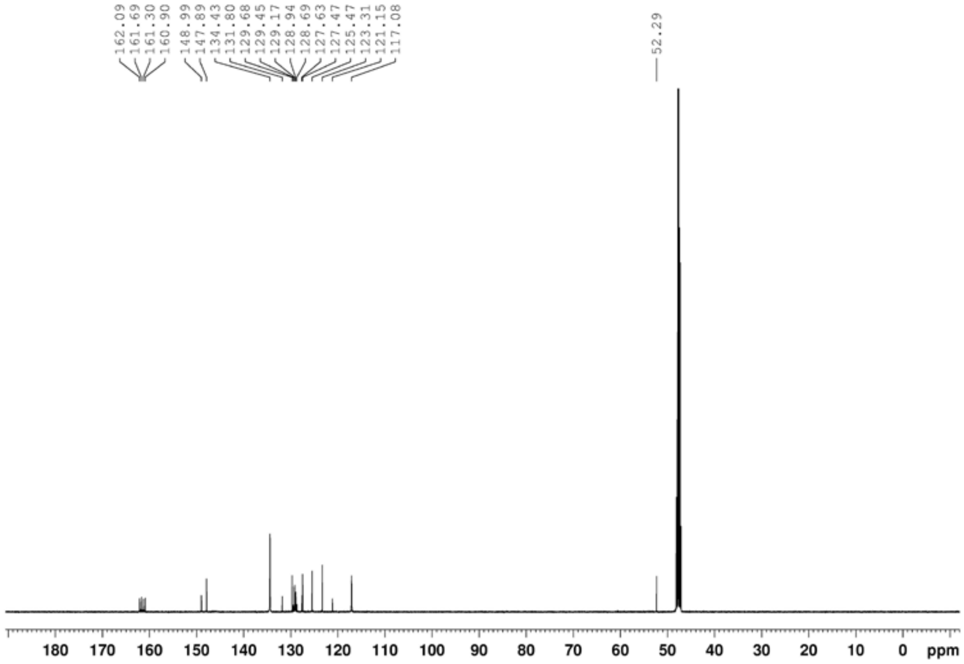

Figure S37. ^{13}C NMR spectrum (125 MHz) of **10**·2BArF in CD_3OD.

ESI mass spectra of 1•BArF⊂EtBP3 and 1•BArF⊂EtBP4 complexes.

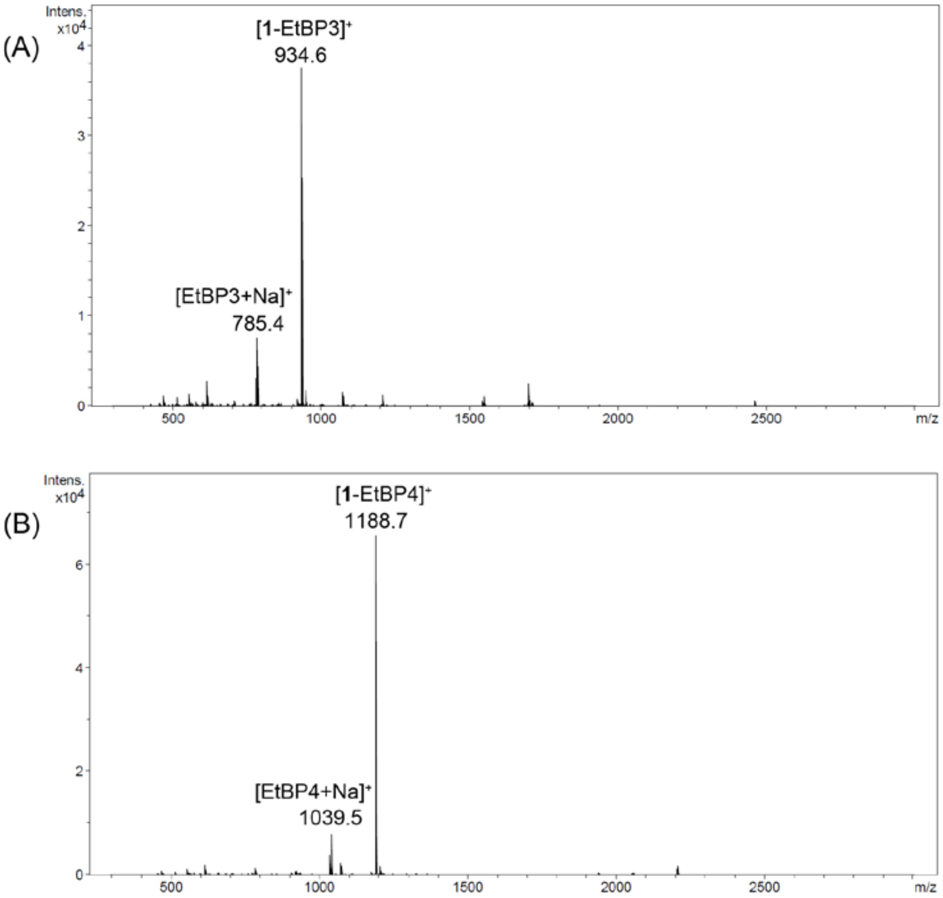

Figure S38. ESI mass spectrum of an equimolar mixture of **1**•BArF−EtBP3 (A) and **1**•BArF−EtBP4 (B) in methanol solution. The concentration of host/guest is about 0.5 μmol L^{-1}.

Job plots.

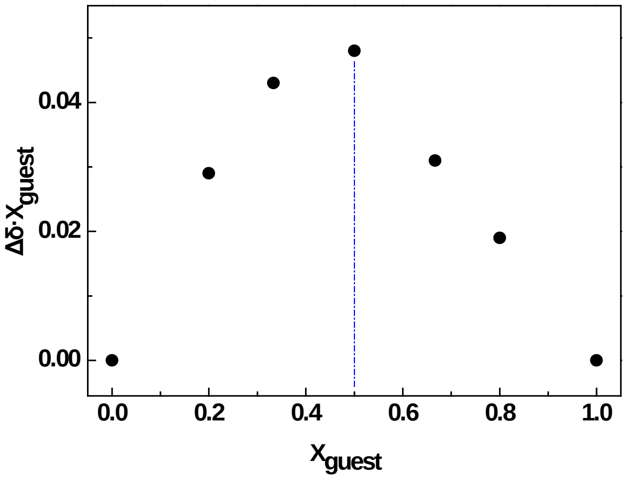

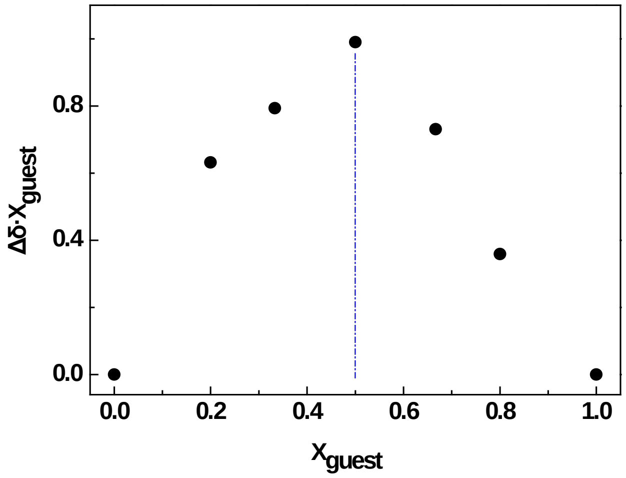

Figure S39. Job plot showing the 1 : 1 stoichiometry of the complexes between **1**•BArF and EtBP3/EtBP4 hosts in $CDCl_3$ by plotting the Δδ in chemical shift of the guest's methyl proton H_a (for proton designations, see Figure 3) observed by ^{1}H NMR spectroscopy against the mole fraction of dimer (X_{host}). Top: **1**•BArF and EtBP3; bottom: **1**•BArF and EtBP4 ([host] + [guest] = 6.0 mM).

^{1}H NMR spectra of guests in the absence and presence of EtBP3 and EtBP4 hosts.

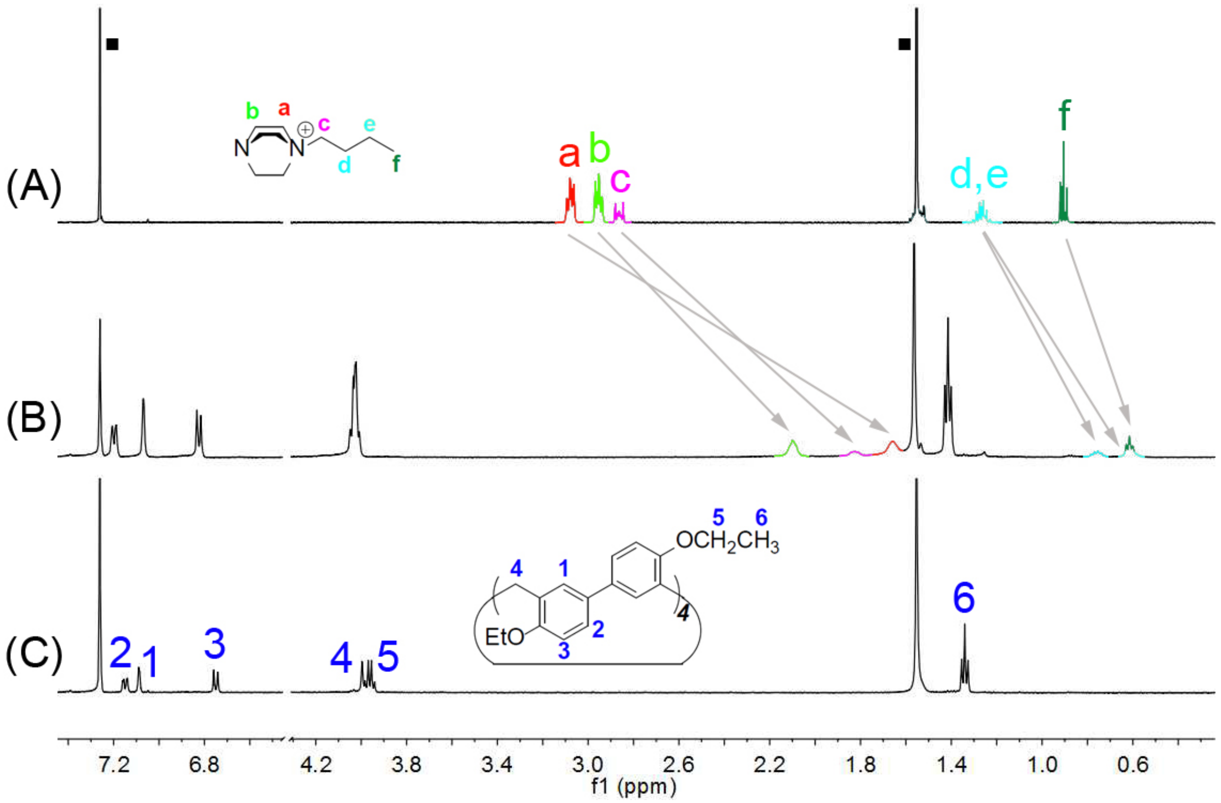

Figure S40. ^{1}H NMR spectra (500 MHz, 298 K) of (A) **5**·BArF, (B) **5**·BArF + EtBP4, and (C) EtBP4 in $CDCl_3$ at 3.0–3.2 mM. "■" = solvent/water.

Figure S40 shows the ^{1}H NMR spectra of **5**·BArF recorded in the absence and in the presence of approximately 1.0 equiv of EtBP4 host. In the presence of EtBP4, the peaks for the guest's protons display substantial upfield shifts (Δδ = −0.29 ~ −1.42 ppm) and broadening effects compared to the free guest as a consequence of inclusion-induced shielding effects, indicating that the host is fully threaded by **5**·BArF guest. Since the Δδ values for H_{a-c} are much larger than those for H_{d-f}, the main binding site for the host is the DBO group.

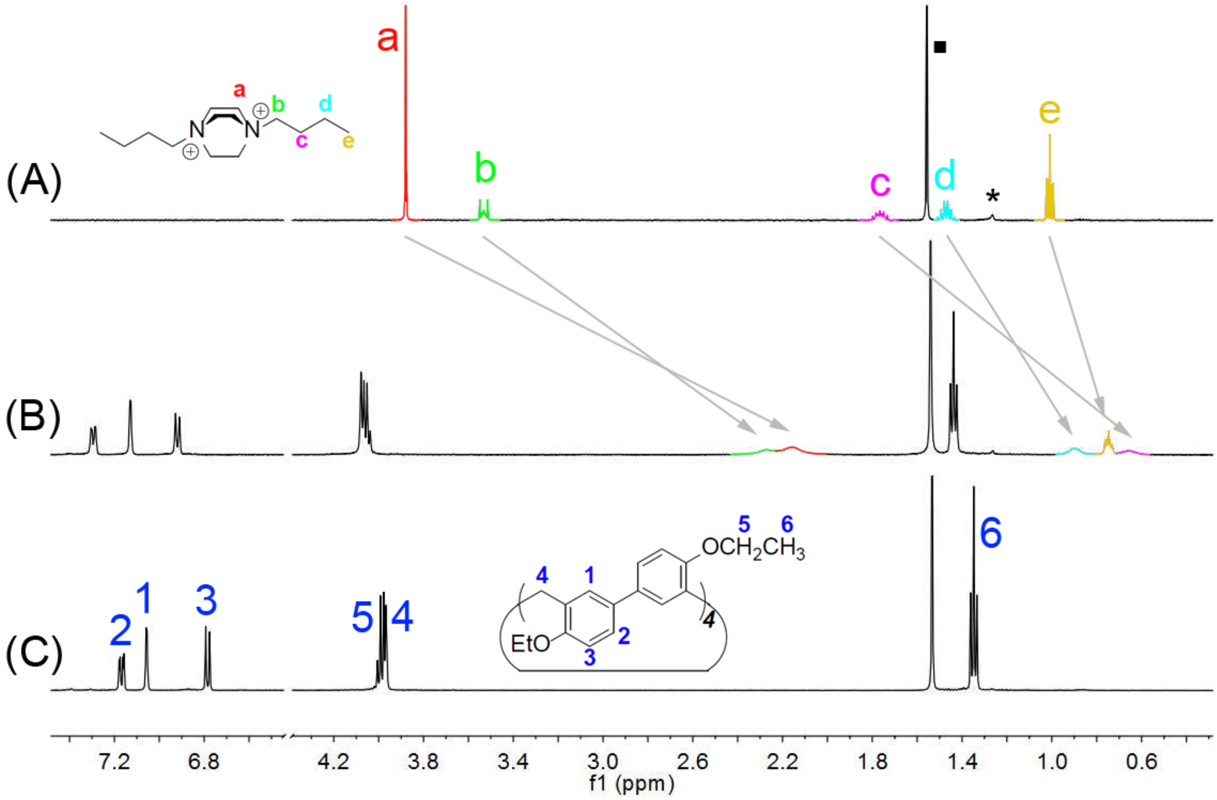

Figure S41. ^{1}H NMR spectra (500 MHz, 298 K) of (A) **6**·2BArF, (B) **6**·2BArF + EtBP4, and (C) EtBP4 in CD_2Cl_2 at 2.9–3.2 mM. "■" = water; "*" = solvent impurities.

Figure S41 shows the ^{1}H NMR spectra of **6**·2BArF recorded in the absence and in the presence of approximately 1.0 equiv of EtBP4 host. In the presence of EtBP4, the peaks for the guest's protons display substantial upfield shifts ($\Delta\delta = -0.26 \sim -1.72$ ppm) and broadening effects compared to the free guest as a consequence of inclusion-induced shielding effects, indicating that the host is fully threaded by **6**·2BArF guest. Since the $\Delta\delta$ values for $H_{a,b}$ are much larger than those for $H_{d,e}$, the main binding site for the host is the central DBO nucleus.

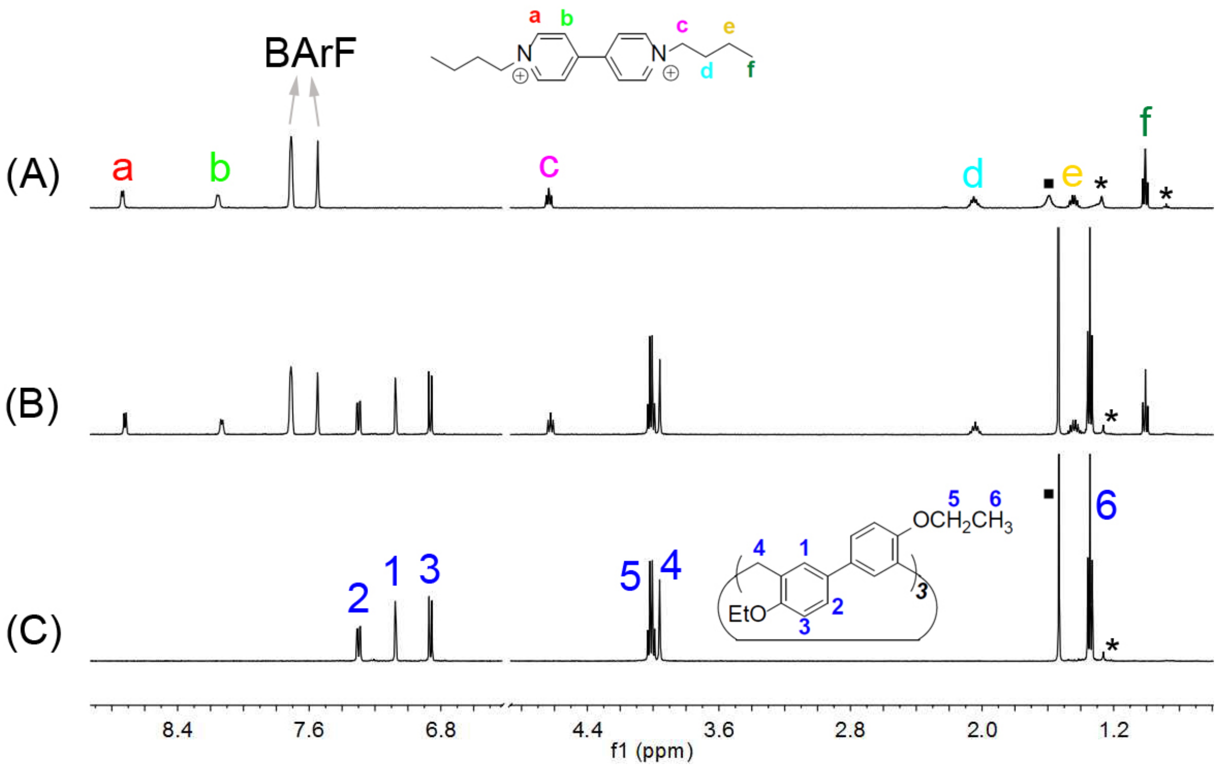

Figure S42. ^{1}H NMR spectra (500 MHz, 298 K) of (A) **7**·2BArF, (B) **7**·2BArF + EtBP3, and (C) EtBP3 in $CDCl_3$ at 3.0–3.3 mM. "■" = water; "*" = solvent impurities.

Figure S42 shows the ^{1}H NMR spectra of **7**·2BArF recorded in the absence and in the presence of approximately 1.0 equiv of EtBP3 host. No obvious signal changes were observed for the guest upon addition of the host, indicating that EtBP3 did not form inclusion complex with **7**·2BArF or at least had very weak interactions.

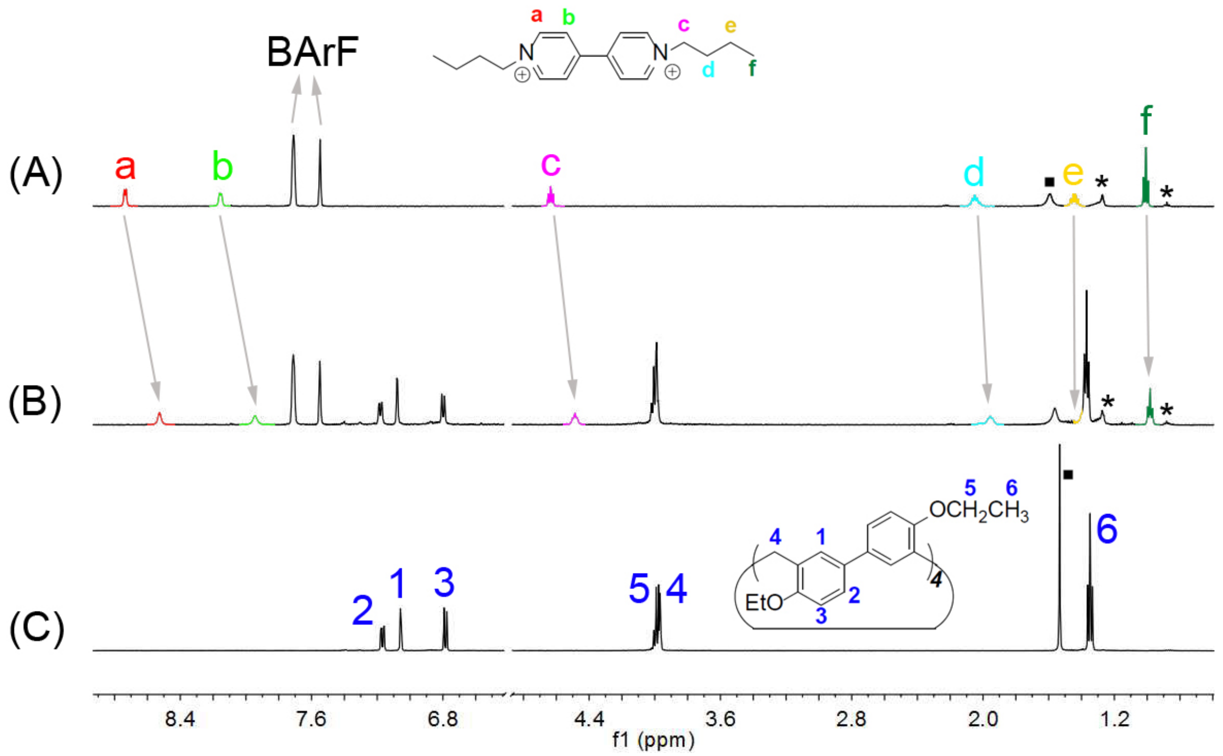

Figure S43. ^{1}H NMR spectra (500 MHz, 298 K) of (A) **7**·2BArF, (B) **7**·2BArF + EtBP4, and (C) EtBP4 in $CDCl_3$ at 2.9–3.1 mM. "■" = solvent/water; "*" = solvent impurities.

Figure S43 shows the ^{1}H NMR spectra of **7**·2BArF recorded in the absence and in the presence of approximately 1.0 equiv of EtBP4 host. In the presence of EtBP4, the peaks for 4,4'-bipyridine protons H_a and H_b and methylene protons H_c display obvious upfield shifts ($\Delta\delta$ = −0.21, −0.21 and −0.15 ppm for H_a, H_b and H_c, respectively) compared to the free guest as a consequence of inclusion-induced shielding effects. At the same time, only small changes can be observed for H_{d-f} protons. These results suggest that the guest's 4,4'-bipyridine unit is deeply included in the cavity of EtBP4.

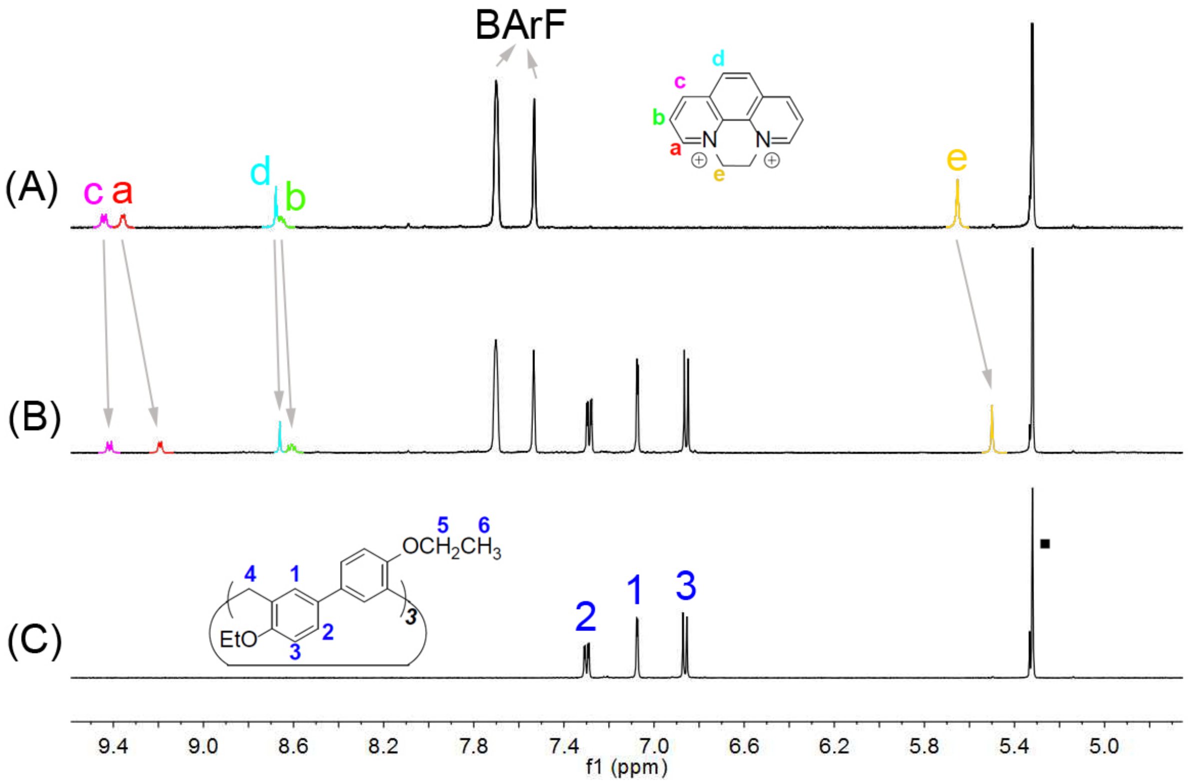

Figure S44. ^{1}H NMR spectra (500 MHz, 298 K) of (A) **10**·2BArF, (B) **10**·2BArF + EtBP3, and (C) EtBP3 in $CDCl_3$ at 3.0–3.1 mM. ▪ = solvent/water.

Figure S44 shows the ^{1}H NMR spectra of **10**·2BArF recorded in the absence and in the presence of approximately 1.0 equiv of EtBP3 host. In the presence of EtBP3, the peaks for amomatic protons H_a and methylene protons H_e display large upfield shifts ($\Delta\delta = -0.17$, and -0.15 ppm for H_a and H_e, respectively), and the H_{b-d} protons show very small NMR changes. These results indicate the formation of a shallow inclusion complex with the guest's "$^+N(CH_2)_2N^+$" site; this binding mode is similar with those for quaternary ammonium guests **1**•BArF−**4**•BArF.

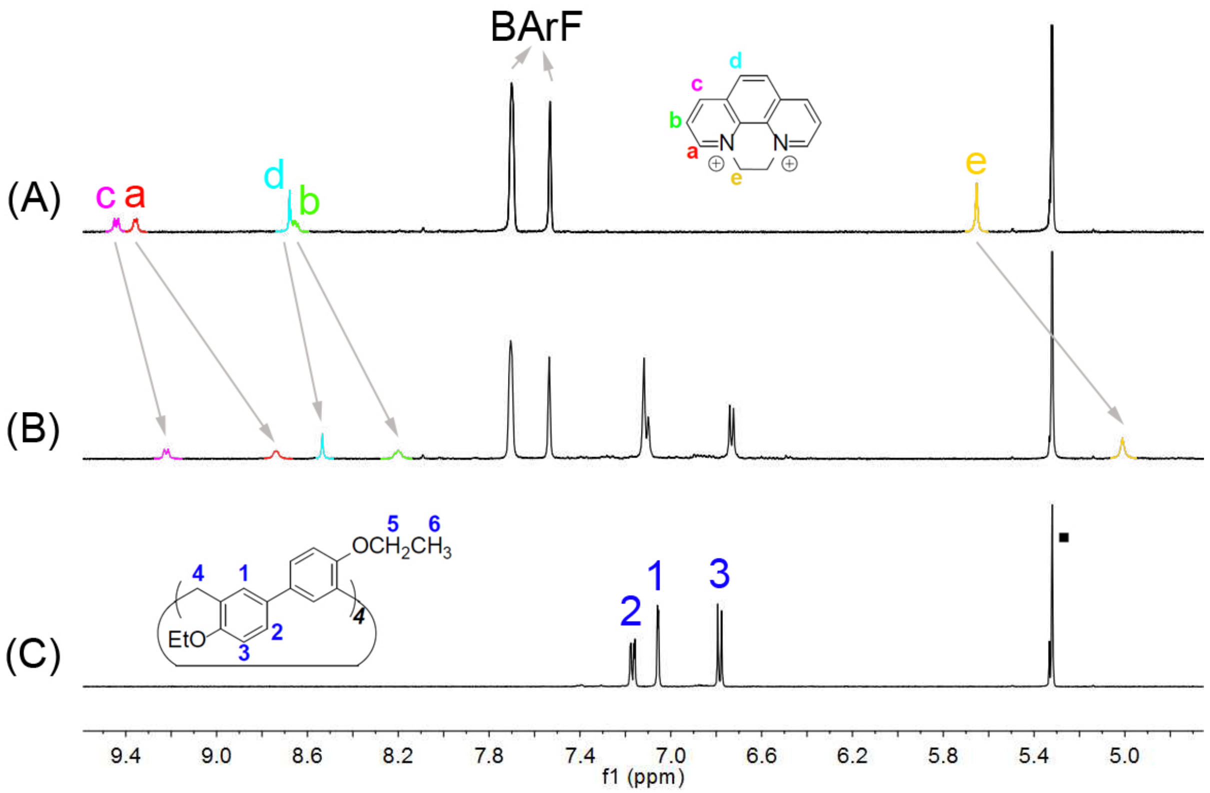

Figure S45. ^{1}H NMR spectra (500 MHz, 298 K) of (A) **10**·2BArF, (B) **10**·2BArF + EtBP4, and (C) EtBP4 in $CDCl_3$ at 2.9–3.2 mM. ▪ = solvent/water.

It is found that in the presence of EtBP4, proton signals of **10**·2BArF exhibit pronounced upfield displacement (Δδ = −0.15 ~ −0.65 ppm) as a consequence of inclusion-induced shielding effects (Figure S45). These results are consistent with the formation of an interpenetrated complex.

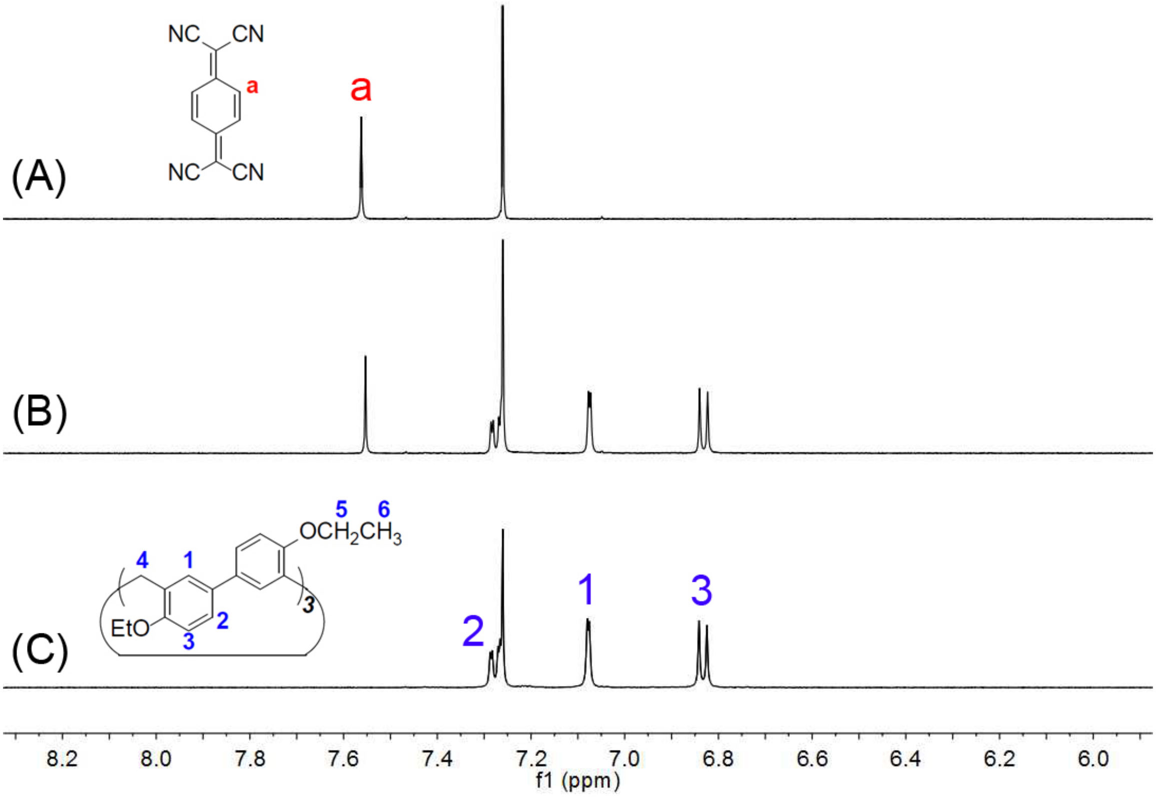

Figure S46. ^{1}H NMR spectra (500 MHz, 298 K) of (A) **11**·2BArF, (B) **11**·2BArF + EtBP3, and (C) EtBP3 in $CDCl_3$ at 3.0–3.3 mM.

Figure S46 shows the ^{1}H NMR spectra of guest **11** recorded in the absence and in the presence of approximately 1.0 equiv of EtBP3 host. No obvious signal changes were observed for the guest upon addition of the host, indicating that EtBP3 did not form inclusion complex with **11** or at least had very weak interactions.

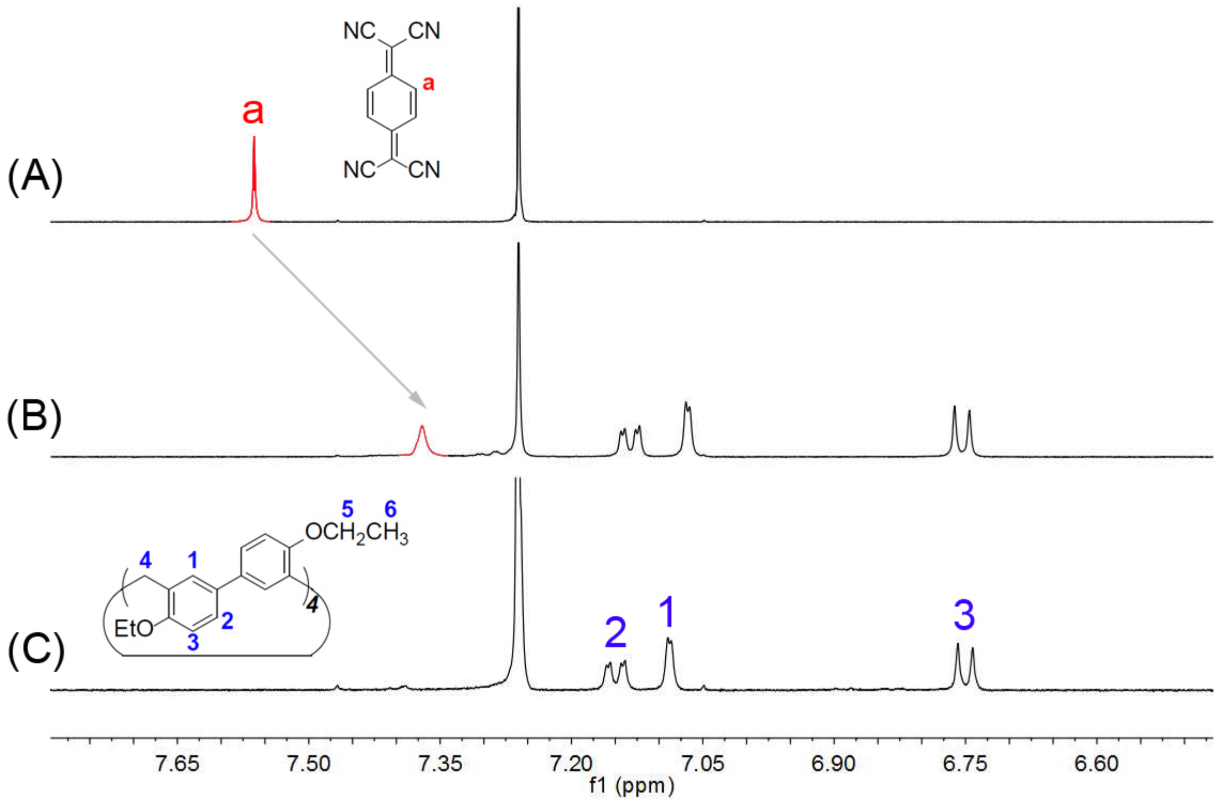

Figure S47. ^{1}H NMR spectra (500 MHz, 298 K) of (A) **11**·2BArF, (B) **11**·2BArF + EtBP4, and (C) EtBP4 in $CDCl_3$ at 3.0–3.1 mM.

Figure S47 shows the ^{1}H NMR spectra of **11**·2BArF recorded in the absence and in the presence of approximately 1.0 equiv of EtBP4 host. In the presence of EtBP4, the peaks for amomatic protons H_a of the guest display substantial upfield shifts ($\Delta\delta = -0.19$ ppm) and broadening effects compared to the free guest as a consequence of inclusion-induced shielding effects, indicating that the host engulfs the guest to form stable inclusion complex.

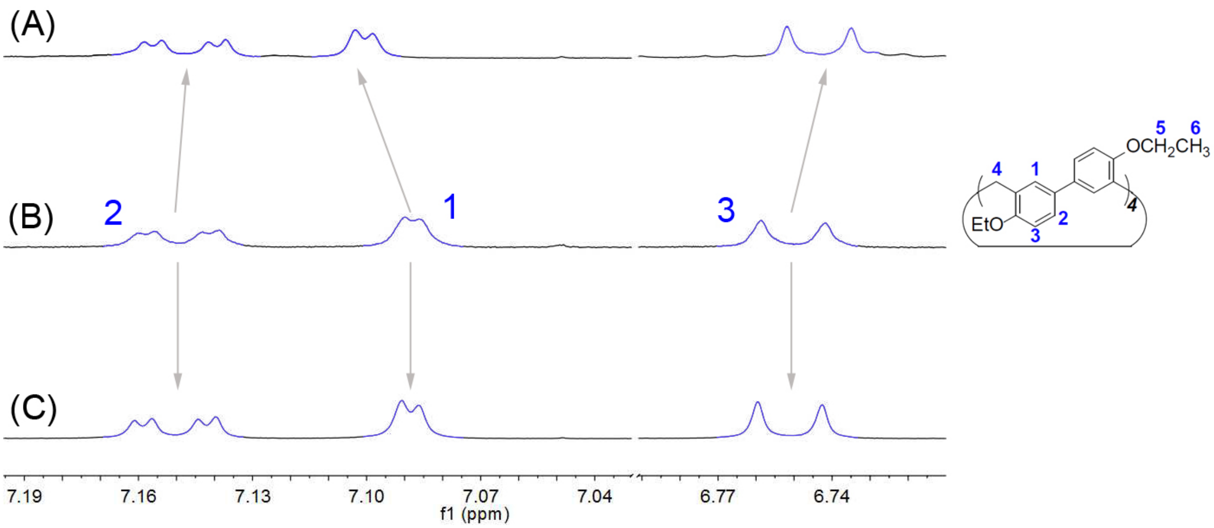

Figure S48. ^{1}H NMR spectra (500 MHz, 298 K) of (A) **12** + EtBP4, (B) EtBP4, and (C) **13** + EtBP4 in $CDCl_3$ at 3.0–3.3 mM.

Although **12** and **13** do not have proton signals, their complexation behavior with EtBP4 can be examined according to the signal changes of the host. Figure S48 shows the ^{1}H NMR spectra of EtBP4 recorded in the absence and in the presence of approximately 1.0 equiv of guests **12** and **13**. No obvious signal changes were observed for the host upon addition of **13** (Figure S48C), indicating no complexation between EtBP4 and **13**. While for EtBP4–**12** pair, the complexation can be detected (Figure S48A) and the association constant is 100 ± 20 M^{-1} by employing ^{1}H NMR titration experiments.

EtBP3 or EtBP4 cannot form complexes with neutral guests **14**–**18**, since no obvious NMR changes can be observed when mixing these host-guest pairs. Figure S49 and S50 show the ^{1}H NMR spectra of **14** and **17** recorded in the absence and in the presence of approximately 1.0 equiv of EtBP4 host.

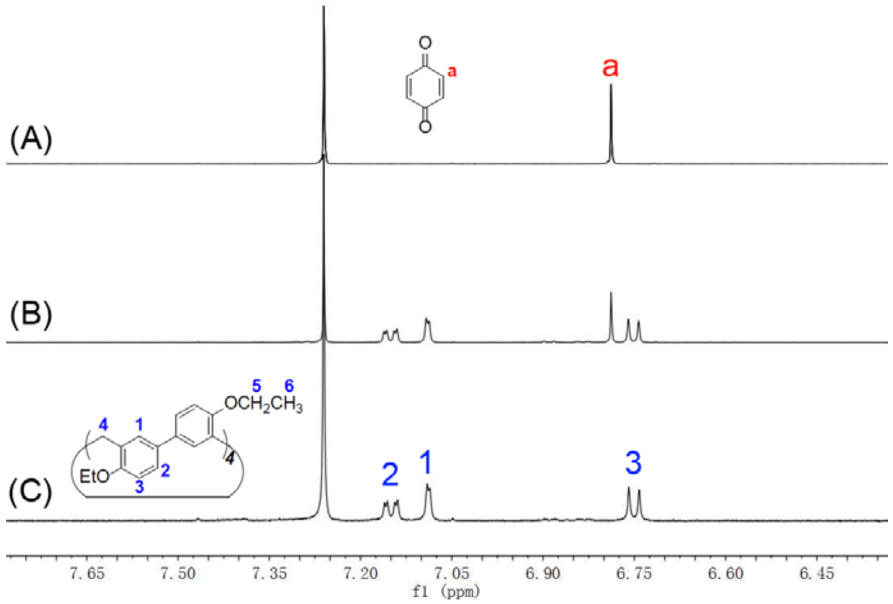

Figure S49. ^{1}H NMR spectra (500 MHz, 298 K) of (A) **14**, (B) **14** + EtBP4, and (C) EtBP4 in $CDCl_3$ at 3.0–3.3 mM. ▪ = solvent/water.

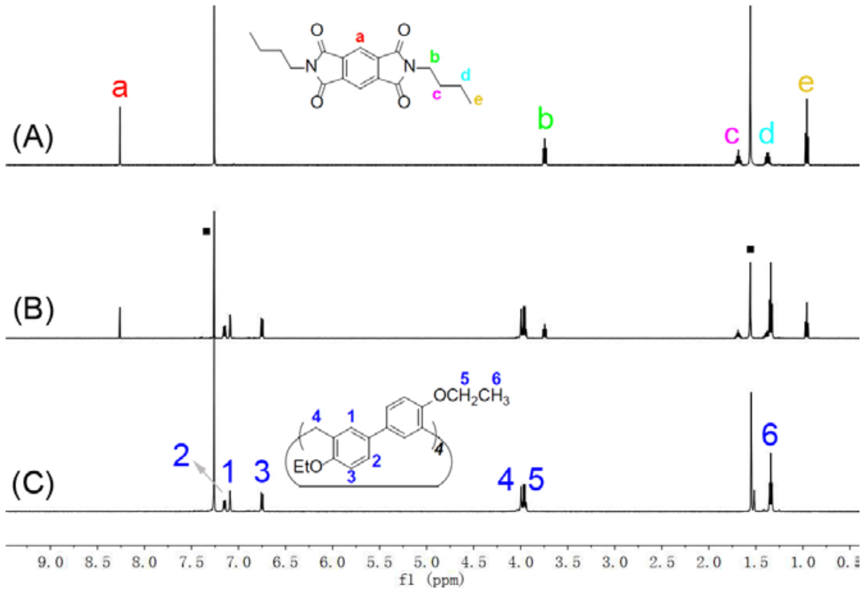

Figure S50. ^{1}H NMR spectra (500 MHz, 298 K) of (A) **17**, (B) **17** + EtBP4, and (C) EtBP4 in $CDCl_3$ at 2.9–3.1 mM. "■" = solvent/water.

Determination of the association constants.

For all the host−guest pairs, chemical exchange is fast on the NMR time scale. To determine the association constant, NMR titrations were done with solutions which had a constant concentration of biphenarene host and varying concentrations of guest. Using the nonlinear curve-fitting method, the association constant was obtained for each host-guest combination from the following equation[S4]:

$A = (A_\infty/[H]_0)\ (0.5[G]_0 + 0.5([H]_0+1/K_a)-(0.5\ ([G]_0^2+(2[G]_0(1/K_a - [H]_0)) + (1/K_a + [H]_0)^2)^{0.5}))$

Where A is the chemical shift change of aromatic proton H_3 (for proton designations, see Figure 1 in the manuscript) on biphenarene host at $[G]_0$, A_∞ is the chemical shift change of H_3 when the host is completely complexed, $[H]_0$ is the fixed initial concentration of the biphenarene host, and $[G]_0$ is the initial concentration of guest (Figure S51 and S52). Assuming 1:1 binding stoichiometry between biphenarene host and these guests, the association constants (K_a) could be calculated by using the nonlinear curve-fitting method.[S4] For each host−guest pair examined, the plot of $\Delta\delta$ as a function of $[G]_0$ gave an excellent fit, verifying the validity of the 1:1 binding stoichiometry assumed. Additionally, Job plots also showed the 1:1 complexation stoichiometries (Figure S39).

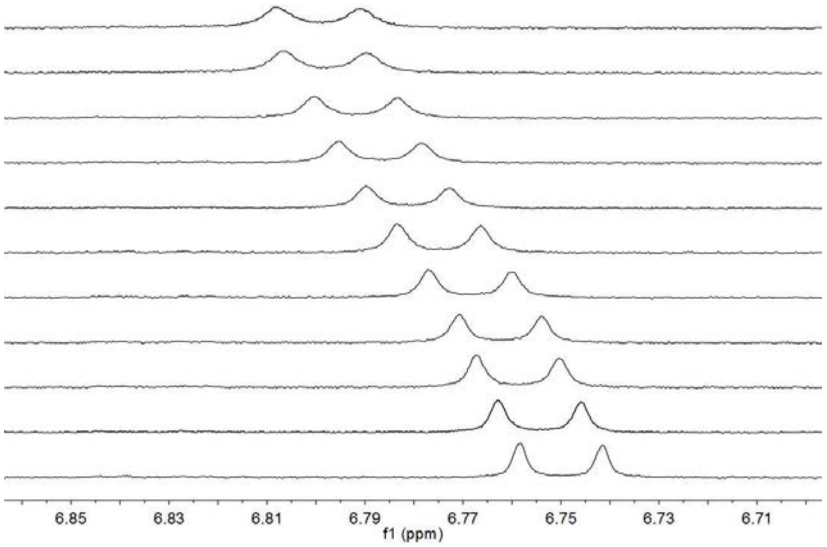

Figure S51. Partial ^{1}H NMR spectra (500 MHz, $CDCl_3$, 298 K) of EtBP4 at a concentration of 4.8×10^{-4} mol/L upon addition of **1**•BArF. From bottom to top, the concentration of **1**•BArF was 0, 0.065, 0.16, 0.25, 0.46, 0.74, 1.1, 1.6, 2.2, 3.2, and 3.7 mM.

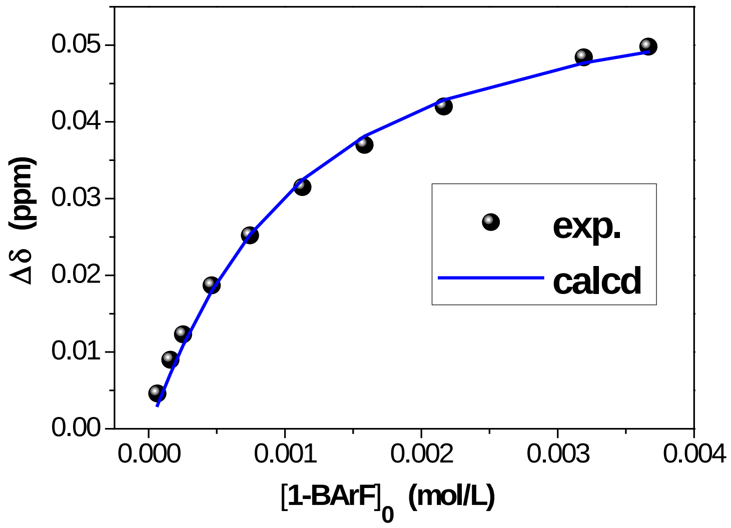

Figure S52. The non-linear curve-fitting (NMR titrations) for the complexation of EtBP4 host (4.8×10^{-4} mol/L) with **1**•BArF in $CDCl_3$ at 298 K.

References.

[S1] N. A. Yakelis, R. G. Bergman *Organometallics*, **2005**, *24*, 3579–3581.

[S2] C. Li, X. Shu, J. Li, J. Fan, Z. Chen, L. Weng, and X. Jia *Org. Lett.*, **2012**, *14*, 4126–4129.

[S3] Swati De, S. Ramakrishnan, Macromolecules 2009, 42, 8599–8603.

[S4] a) K. A. Connors, Binding Constants; Wiley: New York, **1987**; b) R. P. Ashton, R. Ballardini, V. Balzani, M. Belohradsky, M. T. Gandolfi, D. Philp, L. Prodi, F. M. Raymo, M. V. Reddington, N. Spencer, J. F. Stoddart, M. Venturi , D. J. Williams, *J. Am. Chem. Soc.*, **1996**, *118*, 4931–4951; c) Y. Inoue, K. Yamamoto, T. Wada, S. Everitt, X.-M. Gao, Z.-J. Hou, L.-H. Tong, S.-K. Jiang, H.-M. Wu, *J. Chem. Soc., Perkin Trans. 2*, **1998**, 1807−1816; d) X. Shu, S. Chen, J. Li, Z. Chen, L. Weng, X. Jia, C. Li, *Chem. Commun.*, **2012**, *48*, 2967–2969.
